# Genetic diversity, population structure, and benzimidazole resistance profiling of *Ascaris lumbricoides* in northwestern Thailand

**DOI:** 10.1007/s00436-026-08727-w

**Published:** 2026-07-20

**Authors:** Poom Adisakwattana, Tippayarat Yoonuan, Dorn Watthanakulpanich, Patsakorn Boonkead, Kateisara Phaiphilai, Orawan Phuphisut

**Affiliations:** 1 Department of Helminthology, Faculty of Tropical Medicine, Mahidol University, Bangkok, 10400 Thailand; 2 Ministry of Public Health, Chiang Mai Provincial Public Health Office, Communicable Disease Control, Chiang Mai, 50180 Thailand

**Keywords:** *Ascaris lumbricoides*, *Ascaris suum*, Genetic diversity, Population genetics, Mitochondrial DNA, Benzimidazole resistance, *β-tubulin*, *cox1*, *nad1*

## Abstract

*Ascaris lumbricoides* remains one of the most prevalent soil-transmitted helminths worldwide, with ongoing transmission in endemic regions despite long-standing control programs. This study investigated the genetic diversity, population structure, and benzimidazole resistance of *A. lumbricoides* across three endemic regions of Thailand using mitochondrial (*cox1* and *nad1*) and nuclear (*β-tubulin*) markers. Haplotype network analysis identified 13 haplotypes within the Thailand dataset, including three newly identified haplotypes, indicating measurable genetic diversity at the local scale. When analyzed in a global context incorporating reference sequences, substantially higher haplotype diversity was observed, reflecting broad genetic diversity across geographic regions. Despite this diversity, no clear geographic structuring was detected among Thai populations, suggesting ongoing gene flow and population connectivity. Phylogenetic analyses further demonstrated that all isolates clustered within a single major lineage and shared haplotypes with *Ascaris suum* (infecting pig), supporting genetic overlap and potential zoonotic transmission. Analysis of the *β-tubulin* gene revealed exclusively wild-type alleles at codons 167, 198, and 200, with no evidence of mutations associated with benzimidazole resistance. Overall, this study demonstrates measurable genetic diversity in Thailand’s *A. lumbricoides* populations, with no evidence of clear geographic structuring. These findings provide baseline molecular data to support future surveillance of transmission dynamics and anthelmintic resistance.

**Supplementary Information:**

The online version contains supplementary material available at https://doi.org/10.1007/s00436-026-08727-w.

## Introduction

*Ascaris lumbricoides*, the largest intestinal nematode infecting humans, remains one of the most prevalent soil-transmitted helminths worldwide (Centers for Disease Control and Prevention 2026). A recent systematic review estimated that approximately 732 million people were infected globally between 2010 and 2021, with the highest prevalence observed among children (13.5%) and rural populations (17.33%). Southeast Asia ranked as the second highest-prevalence region (19.06%), while the pooled prevalence in Thailand was estimated at 3.62% (Holland et al. 2022).

Although national control programmes have substantially reduced soil-transmitted helminth infections, a 2019 nationwide survey in Thailand still reported detectable *A. lumbricoides* infections across multiple regions, with prevalence ranging from 0.02% in the North to 0.13% in the South (Wattanawong et al. 2021). Importantly, ascariasis remains endemic in certain rural communities, including among Karen hill tribe children in Chiang Mai Province (13%) (Yanola et al. 2018) and in Tak Province (7.78%) (Adisakwattana et al. 2020). These findings indicate that transmission persists in localized settings, particularly in areas with inadequate sanitation, despite long-standing control efforts.

The selected study regions represent geographically distinct settings, ranging from border communities with moderate accessibility to remote mountainous areas. Despite these topographic differences, the communities share epidemiological characteristics relevant to *Ascaris* transmission. The study populations predominantly comprise ethnic minority groups, among whom pigs are commonly raised at the household level for consumption and traditional cultural and ceremonial practices. In households that keep pigs, animals are often maintained in close proximity to human dwellings and allowed to roam freely within the village environment. Furthermore, challenges related to sanitation and hygiene remain common. These shared ecological and epidemiological characteristics provide a strong rationale for selecting these sites to investigate the transmission dynamics and potential zoonotic overlap of *A. lumbricoides.*

Beyond epidemiological monitoring, molecular characterization of parasite populations is essential for understanding transmission dynamics, population structure, and potential zoonotic transmission. A range of genetic markers—including nuclear ITS regions and mitochondrial genes such as *cox1*, *cox2*, *nad1*, and *cytb*—have been used to investigate genetic variation in *A. lumbricoides* and *Ascaris suum* (Peng et al. 2005; Leles et al. 2010; Betson et al. 2011, 2014; Cavallero et al. 2013; Sadaow et al. 2018; Monteiro et al. 2019; Eamsobhana et al. 2022). These studies have demonstrated substantial genetic overlap between the two taxa, supporting a shared evolutionary background and raising questions regarding species boundaries and cross-host transmission (Criscione 2013).

In Thailand, previous molecular investigations based on ITS regions and mitochondrial *cox1* sequences confirmed the presence of *Ascaris* spp., identified novel haplotypes, and suggested possible hybrid genotypes between human- and pig-derived worms (Sadaow et al. 2018). Subsequent analyses incorporating multiple mitochondrial loci revealed complex phylogenetic patterns and the presence of major mitochondrial clades shared across host species (Eamsobhana et al. 2022). However, these studies were limited by relatively small sample sizes, constraining inference regarding population structure within the country.

Control of ascariasis in Thailand relies primarily on periodic mass drug administration (MDA) using benzimidazole anthelmintics. Although these drugs remain broadly effective, extensive evidence from veterinary helminths indicates that repeated benzimidazole exposure can select for resistant parasite populations (Kwa et al. 1994; Prichard 2001; Wolstenholme et al. 2004; Ghisi et al. 2007; Sutherland and Leathwick 2011). Resistance has been associated with single nucleotide polymorphisms at codons 167, 198, and 200 of the *β-tubulin* gene (Jones et al. 2022). Several studies have investigated benzimidazole resistance-associated mutations, with some reporting their presence in regions including Haiti, Kenya, Panama, and Brazil (Diawara et al. 2009, 2013; Krücken et al. 2017; Rashwan et al. 2017; Zuccherato et al. 2018; Furtado et al. 2019; Matamoros et al. 2019; Roose et al. 2021). To date, systematic molecular surveillance of these resistance-associated polymorphisms has not been conducted in Thailand.

Given the persistence of localized endemic foci, the documented mitochondrial variation of *Ascaris* spp., and the absence of integrated resistance monitoring, further comprehensive investigation of *A. lumbricoides* populations in Thailand is warranted. The present study aimed to characterize genetic diversity and population structure of *A. lumbricoides* across three endemic regions of Thailand using mitochondrial *cox1* and *nad1* markers. In addition, the *β-tubulin* gene was analyzed to screen for known benzimidazole resistance–associated polymorphisms. By integrating mitochondrial diversity analysis with resistance screening, this study provides baseline molecular data to support future surveillance of transmission dynamics and anthelmintic resistance in Thailand.

## Materials and methods

### Ethics approval and consent to participate

This study has been approved by the Human Research Ethics Committee of the Faculty of Tropical Medicine, Mahidol University, Bangkok, Thailand. Certificate no: MUTM 2024–080–01 and MUTM 2024–080–02. In addition, partial *A. lumbricoides* adult samples were obtained from a previously archived samples approved by the Ethics Committee of the Faculty of Tropical Medicine, Mahidol University (Certificate no: MUTM 2021–020–02).

This study has been approved by the Faculty of Tropical Medicine – Institutional Animal Care and Use Committee (FTM-IACUC) Mahidol University, Bangkok, Thailand. Certificate no: FTM – ACUC 026/2024E.

This study has been approved by Institutional Biosafety Committee, Faculty of Tropical Medicine, Mahidol University, Bangkok, Thailand. Certificate no: TM 2024–024 and TM 2026–004.

### Study areas

Adult worm samples from Tha Song Yang District, Tak Province, were obtained from previously archived samples (Certificate of Ethical Approval No. MUTM 2021–020-02). Adult worms were subsequently obtained from participants residing in Umphang District, Tak Province, and Omkoi District, Chiang Mai Province. The collection procedure involved the examination of fecal samples and the retrieval of adult worms from *A. lumbricoides*-infected participants. The target population comprised school-age children residing in Umphang District, Tak Province, and Omkoi district, Chiang Mai Province. The study was conducted at Subdistrict Health Promotion Hospitals in Umphang District, Tak Province, and Omkoi district, Chiang Mai Province.

### Sample collection

Fecal examinations were conducted in the study areas to identify *A. lumbricoides* infections. Fecal samples were collected from participants who met the inclusion criteria. Each participant received a standard fecal collection container labeled with their unique code and equipped with a plastic spoon for sample collection. The collected fecal samples were delivered to the laboratory on the same day of collection. Fecal samples were examined for *A. lumbricoides* infections using the Kato-Katz thick smear technique under a light microscope.

Fecal examinations were conducted following the Kato-Katz thick smear method. Briefly, a small amount of fecal material was placed on absorbent paper and pressed through a wire mesh to obtain a uniform sample. The sieved feces were transferred into the central hole of a template placed on a microscope slide until the hole was completely filled. The template was then removed, and the sample was covered with a cellophane strip pre-soaked in a clearing solution. The preparation was gently pressed with a soft rubber stopper to spread the fecal material into an even layer. The smear was left to stand at ambient temperature for approximately 30 min before being examined under a light microscope. Fecal samples positive for *A. lumbricoides* infection were recorded. All *A. lumbricoides*-positive fecal samples were preserved in 80% ethanol for subsequent analyses.

Participants with *A. lumbricoides*-positive fecal examinations received a single oral dose of 400 mg albendazole (According to the World Health Organization (WHO) Guideline: Preventive chemotherapy to control soil-transmitted helminth infections in at-risk population groups, single dose albendazole (400 mg) is recommended for deworming in young children, preschool and school-age children) (World Health Organization 2026), administered by a physician. Following treatment, participants were asked to voluntarily collect whole day fecal sample for four consecutive days. Expelled adult *A. lumbricoides* worm were collected and subsequently processed by washing three times with Millipore’s high-quality ultrapure water (Milli-Q water). The specimens were then preserved in 80% ethanol for further analysis.

### DNA extraction from adult worms

Genomic DNA (gDNA) was extracted from adult worm tissue samples using the Tissue Genomic DNA Mini Kit (Geneaid, Taoyuan, Taiwan), following the manufacturer’s instructions. The quality and quantity of the extracted gDNA were assessed by agarose gel electrophoresis and a Nanodrop spectrophotometer, respectively. The purified gDNA was stored at − 20 °C for subsequent analyses.

### Isolation and purification of Ascaris eggs

When adult worms were not available, eggs recovered from fecal samples were used as the DNA source. Eggs were isolated by sucrose flotation (specific gravity 1.27) following a protocol modified from Fausto et al. (2021). Briefly, approximately 2 g of fecal sample was homogenized in sucrose solution, filtered through gauze to remove large debris, and subjected to flotation. The supernatant fraction containing eggs was collected, and eggs were identified microscopically and transferred to 1.5-mL microcentrifuge tubes. To reduce fecal contamination and remove potential potential polymerase chain reaction (PCR) inhibitors, the recovered eggs were repeatedly washed with sterile Milli-Q water and centrifuged until the supernatant became clear and free of visible debris. The final egg pellet was resuspended in sterile Milli-Q water and stored at − 20 °C prior to DNA extraction.

### Single-egg preparation for PCR

Individual eggs were examined under a stereomicroscope and mechanically disrupted using a sterile fine needle to rupture the eggshell. The released material from a single egg was used directly as the PCR template. Each reaction contained DNA derived from one egg to ensure independent genotyping.

### *Cox1* and *nad1* gene amplification and sequencing

Partial fragments of *cox1* (~ 431 bp) and *nad1* (~ 400 bp) genes were amplified as previously described (Peng et al. 2005). The primer pairs used were as follows: for *cox1*, forward 5′-TTTTTTGGTCATCCTGAGGTTTAT-3′ and reverse 5′-ACATAATGAAAAT-GACTAACAAC-3′; for *nad1*, forward 5′-TATGAGCGTCATTTATTGGG-3′ and reverse 5′-GCAT-CACAATAGCCAACAAATAC-3′.

PCR reactions for adult worms were performed in a final volume of 50 μL containing 1 × KAPA2G Fast HS ReadyMix with dye (Kapa Biosystems, Roche; final concentration 1.5 mM MgCl₂ and 0.2 mM of each dNTP), 0.2 μM of each forward and reverse primer, 50 ng of gDNA template, and Milli-Q water to volume. For single eggs, reactions were performed in a 50 μL volume containing 1.25 U TaKaRa Ex Taq® DNA Polymerase (TaKaRa Bio Inc., Japan), 1 × Ex Taq buffer, 0.2 mM of each dNTP, 0.4 M betaine, 0.2 μM of each primer, and a mechanically disrupted single egg as template.

Thermal cycling for *cox1* consisted of an initial denaturation at 94 °C for 5 min, followed by 35 cycles of 94 °C for 30 s, 55 °C for 30 s, and 72 °C for 45 s, with a final extension at 72 °C for 5 min. For *nad1*, cycling conditions included an initial denaturation at 94 °C for 3 min, followed by 35 cycles of 94 °C for 30 s, 55 °C for 30 s, and 72 °C for 60 s, with a final extension at 72 °C for 5 min. A non-template control (NTC) was included in each PCR run.

PCR amplicons were separated on 1% agarose gels prepared in 0.5 × Tris–borate–EDTA (TBE) buffer and electrophoresed at 100 V for approximately 35 min. Bands were visualized using a Gel Doc XR + Gel Documentation System (Bio-Rad Laboratories, Inc., USA). PCR products were purified prior to DNA sequencing. Chromatograms were manually inspected. Sequence identities were confirmed using BLASTn against the NCBI nucleotide database.

### *β-tubulin* gene amplification and sequencing

Amplification of the *β-tubulin* gene targeting codons 167, 198, and 200 was performed using a semi-nested PCR approach as previously described (Monteiro et al. 2019). For codon 167, the first-round PCR employed primers F1-167 (5′-TCCGTGAAGAATACCCCGACA-3′) and R1-167 (5′-CAACCAACTGATGAACGGAC-3′), generating a 398 bp fragment. The second round used primers F1-167 and R2-167 (5′-CCACACTTGAACCTGCTAACG-3′), yielding a 129 bp amplicon. For codons 198 and 200, the first-round PCR used primers F1 198/200 (5′-AGAGCCACAGTTGGTTTAGA-TACG-3′) and R1 198/200 (5′-AGGGTCCTGAAGCAGATGTC-3′), producing a 191 bp fragment. The second round employed primers F1 198/200 and R2 198/200 (5′-CAGATGTCGTACAAAGCCTCATT-3′), resulting in a 179 bp amplicon.

PCR amplification for adult worms was performed in a final volume of 50 μL containing 1.25 U TaKaRa Ex Taq® DNA Polymerase (TaKaRa Bio Inc., Japan), 1 × Ex Taq buffer, 0.2 mM of each dNTP, 0.4 M betaine, 0.2 μM of each primer, and 50 ng of gDNA template, with Milli-Q water added to adjust the final volume. For single eggs, reactions were performed under the same conditions, except that a mechanically disrupted single egg was used as the DNA template in place of extracted gDNA.

Thermal cycling consisted of an initial denaturation at 94 °C for 3 min, followed by 35 cycles of denaturation at 94 °C for 45 s, annealing at 59 °C for 45 s, and extension at 68 °C for 1 min, with a final extension at 68 °C for 5 min. A non-template control (NTC) was included in each PCR run. PCR products were analyzed and sequenced as described above for mitochondrial genes.

### Data analysis

The obtained *cox1* and *nad1* sequences were aligned and analyzed to assess genetic variation. In addition, publicly available *Ascaris* spp. sequences were retrieved from the NCBI GenBank database and incorporated into the dataset for comparative analysis. Haplotype diversity indices, including the number of haplotypes (H), haplotype diversity (Hd), nucleotide diversity (π), and the average number of nucleotide differences (k), were calculated using DnaSP v5 (Librado and Rozas 2009). Haplotype networks were constructed using the median-joining algorithm implemented in PopART (Bandelt et al. 1999).

Phylogenetic relationships were inferred using the Maximum Likelihood (ML) method implemented in MEGA X software (Kumar et al. 2018). The best-fit nucleotide substitution model was selected based on the Bayesian Information Criterion (BIC) in MEGA X prior to tree reconstruction. Branch support was assessed using 1,000 bootstrap replicates. The resulting phylogenetic trees were visualized and edited using FigTree v1.4.4 (Rambaut 2018). Sequence alignments were manually inspected and edited using BioEdit software (Hall 1999) prior to downstream analyses.

For *β-tubulin*, nucleotide sequences were aligned using MEGA X software. Codon positions 167, 198, and 200 were examined at both the nucleotide and deduced amino acid levels to identify mutation genotypes. Sequences were translated into amino acids within MEGA X to confirm the presence of substitutions associated with benzimidazole resistance. Co-alignment of nucleotide and corresponding amino acid sequences was further visualized using BioEdit software to ensure accurate codon position comparison and genotype determination. The *A. lumbricoides β-tubulin* reference sequence (GenBank accession no. FJ501301.1) was used for positional verification.

## Results

### Sample collection

**Fig. 1:**
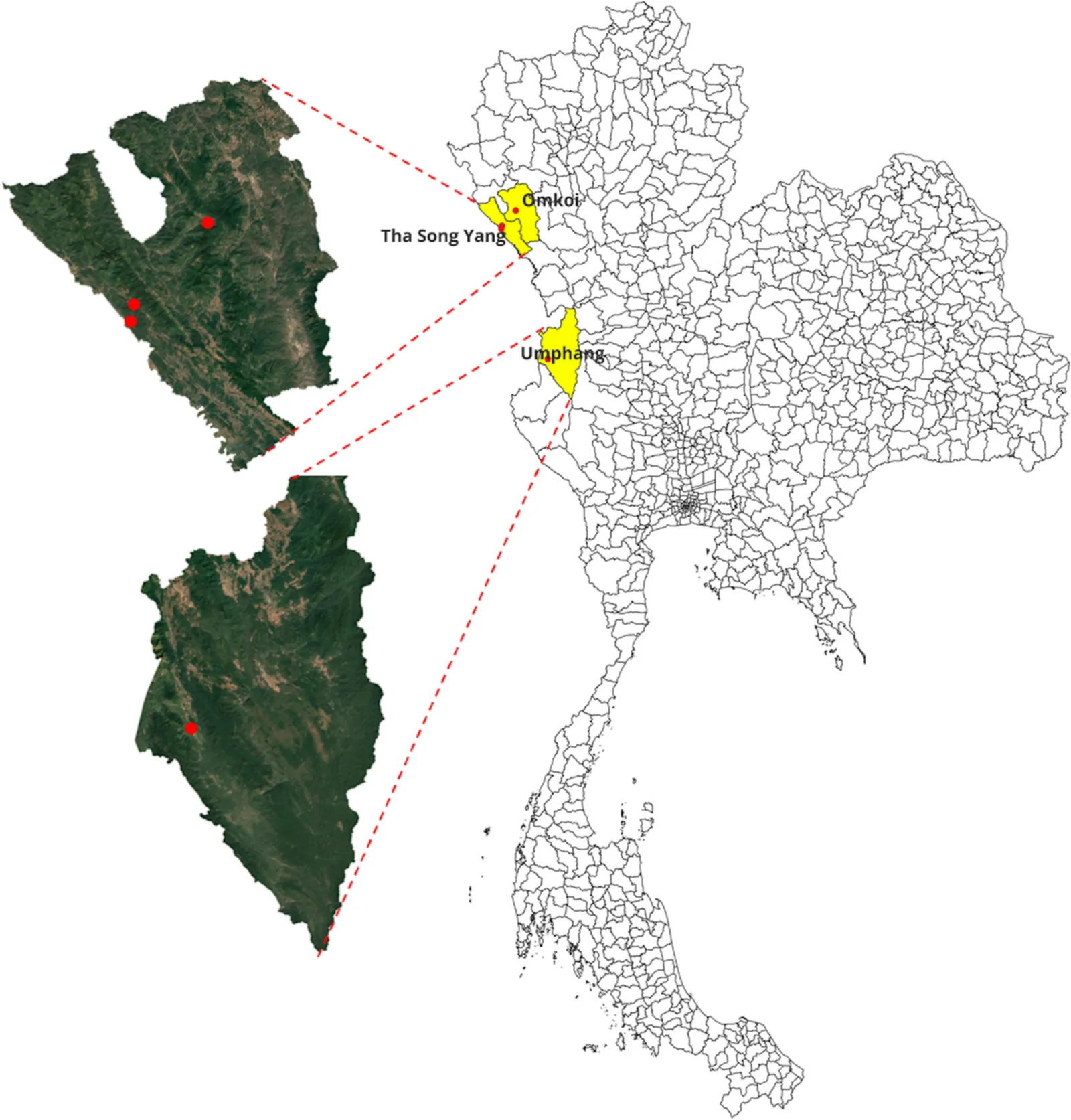
Geographical locations of the study sites. Map indicating collection points for adult worm specimens in Tha Song Yang and Umphang Districts (Tak Province), and Omkoi District (Chiang Mai Province). Map was generated using QGIS v. 3.32 (https://qgis.org) with geographic reference data from Google map. Detailed views of each sampling location are provided in Supplementary Fig. 1

A total of 58 adult worm specimens from Tha Song Yang District, Tak Province were obtained from previously archived samples; these included 15 samples from Greh-kee village and 43 from Mae Salid Luang village. Additional adult worms were collected from infected participants identified during the present study. In total, 171 fecal samples from Umphang District, Tak Province and 125 fecal samples from Omkoi District, Chiang Mai Province were examined. Microscopic examination revealed *A. lumbricoides* infection in 48 samples from Umphang District, Tak Province Province and 10 samples from Omkoi District, Chiang Mai Province (Table 1). Adult worms were successfully recovered from 17 positive cases in Umphang District, Tak Province and 3 positive cases in Omkoi District, Chiang Mai Province, yielding 44 and 4 adult worms, respectively. Overall, 106 adult worm specimens were included in the molecular analyses, comprising samples from Tha Song Yang District, Tak Province (n = 58), Umphang District, Tak Province (n = 44), and Omkoi District, Chiang Mai Province (n = 4) (Table 1). The geographical location of the study sites is shown in Fig. 1.

**Table 1:** Summary of *Ascaris lumbricoides* sample collection across three districts in Tak and Chiang Mai Provinces

Category	Tha Song Yang District, Tak Province	Umphang District, Tak Province	Omkoi District, Chiang Mai Province	Total
Fecal samples examined	–	171	125	296
Fecal samples positive for *A. lumbricoides*	–	48	10	58
Adult worms recovered (cases)	–	17	3	20
Adult worms obtained (specimens)	58	44	4	106
Egg-only cases	–	–	7	7
Eggs isolated	–	–	84	84
Eggs successfully amplified	–	–	53	53

In addition, seven infected cases yielded egg samples only, from which a total of 84 eggs were isolated (12 eggs per case). Of these, 53 eggs were successfully amplified by PCR. Among the successfully amplified egg samples, 17, 14, 12, and 10 were amplified for *cox1*, *nad1*, *β-tubulin* codon 167, and *β-tubulin* codons 198 and 200, respectively. Consequently, a total of 123, 120, 118, and 116 samples were included in the final analyses for *cox1*, *nad1*, *β-tubulin* codon 167, and *β-tubulin* codons 198 and 200 (Table 2), respectively.

**Table 2:** Number of samples included in molecular analyses

Gene target	Adult worms	Eggs	Total Analyzed
*cox1*	106	17	123
*nad1*	106	14	120
*β-tubulin* codon 167	106	12	118
*β-tubulin* codons 198 and 200	106	10	116

### PCR amplification and sequencing

1. Mitochondrial *cox1*

Amplification of the mitochondrial *cox1* gene produced a single fragment of approximately 431 bp, as confirmed by agarose gel electrophoresis (Supplementary Fig. 2). Sequences were successfully generated from 123 samples. BLASTn analysis demonstrated 99.73–100% nucleotide identity with reference *A. lumbricoides cox1* sequences (GenBank accession numbers ON725082.1, MK792802.1, and MK792796.1), with E-values of 0 in all comparisons, confirming species identity and correct target amplification.

2. Mitochondrial *nad1*

PCR amplification of the mitochondrial *nad1* gene yielded a single amplicon of approximately 400 bp (Supplementary Fig. 3). Sequences were successfully generated from 120 samples. BLASTn comparison showed 99.70% nucleotide identity with reference *A. lumbricoides nad1* sequences (GenBank accession numbers MK160535.1, MK160532.1, and MK160534.1), with an E-value of 3 × 10^–170^, confirming amplification of the intended locus.

3.  *β-tubulin* (codon 167)

First-round PCR targeting *β-tubulin* codon 167 generated a 389 bp fragment (Supplementary Fig. 4A). Nested amplification produced a 129 bp amplicon encompassing the target codon (Supplementary Fig. 4B). Fragment sizes were consistent with expected lengths. Sequences were successfully generated from 118 samples. Sequencing of nested PCR products enabled genotype determination at both the nucleotide and deduced amino acid levels.

4. *β-tubulin* (codons 198 and 200)

For *β-tubulin* codons 198 and 200, primary PCR yielded a 191 bp fragment (Supplementary Fig. 5A), followed by nested amplification producing a 179 bp amplicon covering both codons (Supplementary Fig. 5B). Observed fragment sizes matched expected product lengths. Sequences were successfully generated from 116 samples. The resulting sequences were used for genotype determination at the nucleotide and deduced amino acid levels.

### Haplotype diversity and neutrality analysis of the mitochondrial cox1 gene

Haplotype analysis was performed on 344 *A. lumbricoides cox1* sequences, comprising 123 newly generated sequences from the present study and 221 reference sequences retrieved from public databases (Supplementary Table 1). After alignment and trimming, a 326 bp region was retained for analysis, excluding gaps and missing data.

### Genetic diversity

Across the combined dataset, 86 polymorphic (segregating) sites (S = 86) and 93 total mutations (Eta = 93) were identified. The mean number of nucleotide differences was k = 5.016, and nucleotide diversity was π = 0.01539. A total of 106 haplotypes (h = 106) were detected, yielding a high haplotype diversity (Hd = 0.9262; variance = 0.00006).

In contrast, analysis of the Thailand dataset alone (n = 123) identified 13 haplotypes defined by 11 polymorphic sites (S = 11). Haplotype diversity was moderate (Hd = 0.7827; variance = 0.00055), whereas nucleotide diversity was lower (π = 0.00691), with a mean number of nucleotide differences of k = 2.251.

When Thailand sequences were analyzed together with globally retrieved reference sequences, haplotype richness and overall haplotype diversity increased substantially. However, nucleotide divergence remained relatively low across the full dataset.

### Haplotype network structure

**Fig. 2:**
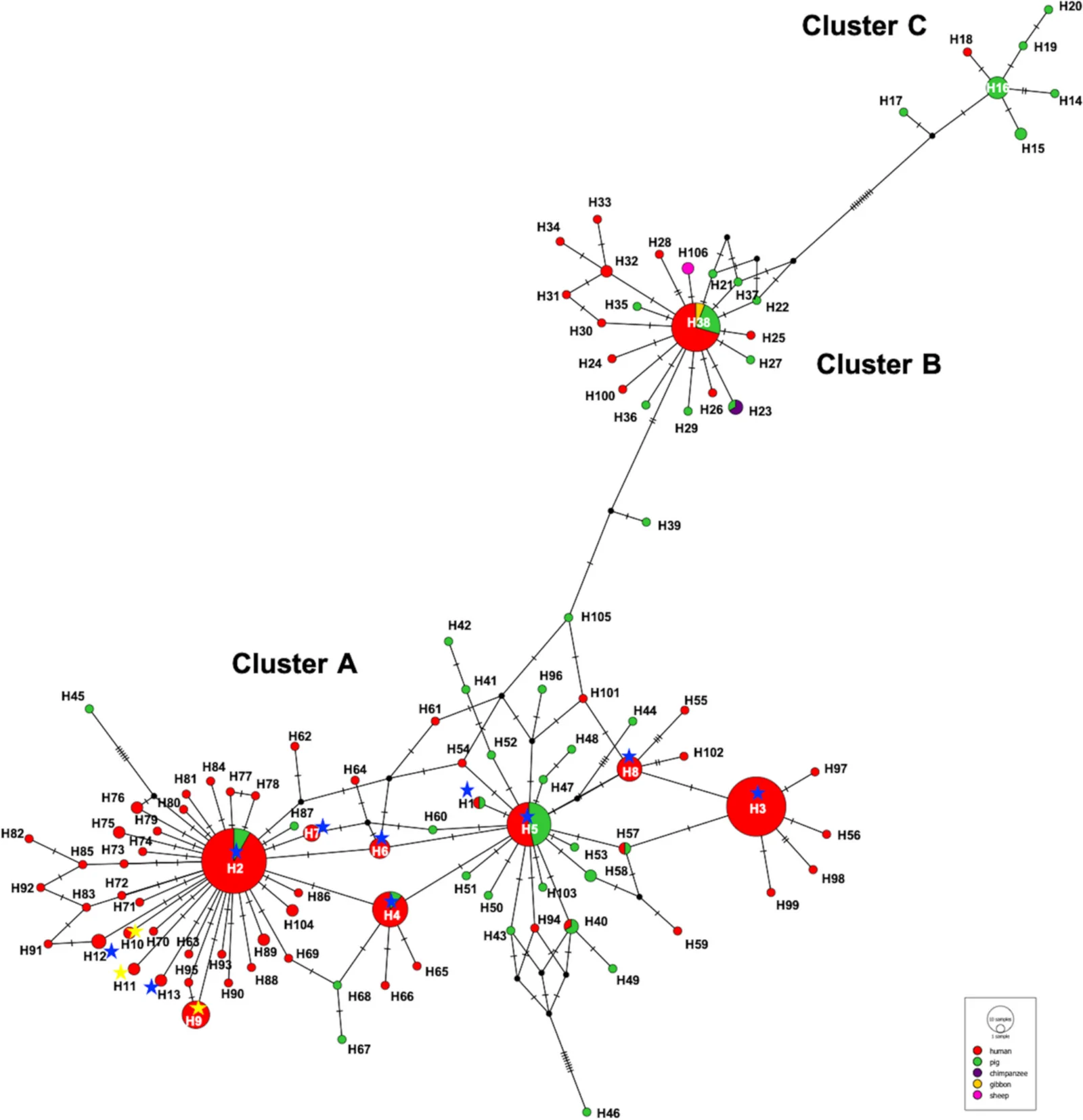
The figure shows the median-joining network of *cox1* sequences of *Ascaris* spp., based on the 123 *Ascaris cox1* sequences obtained in this study and 221 GenBank sequences (n = 344). Blue stars represent samples from this study, while yellow stars denote newly identified haplotypes. Circle size relates to the number of samples with each haplotype

The median-joining haplotype network (Fig. 2) revealed three major clusters (A–C). Most haplotypes were rare, with 80 of 106 (75.5%) represented by single sequences. The most frequent haplotypes were H2 (n = 61), H3 (n = 51), H38 (n = 34), H5 (n = 28), and H4 (n = 18), which together accounted for a large proportion of the total sequences. Additional moderately frequent haplotypes included H9 (n = 11), H8 (n = 9), H16 (n = 7), and H6 (n = 6).

Several common haplotypes were shared across multiple geographic regions, and no distinct clustering by continent or country was observed. The network was characterized by short mutational distances among haplotypes, with many low-frequency and singleton variants radiating from common central haplotypes.

### Haplotype distribution in Thailand samples

Among the 123 Thailand sequences, 13 haplotypes (H1–H13) were identified (Table 3), including three novel haplotypes (H9–H11) detected exclusively in this study. The predominant haplotypes (H2–H5) were shared with isolates from Asia, Africa, Europe and the Americas. For example, haplotype H2 was distributed across Southeast Asia, Africa and South America, whereas H5 was also detected in Europe and North America.

**Table 3:** Distribution of haplotypes across study locations

**Haplotypes**	**Number of haplotypes**				**Total**
	**TGK**	**TSL**	**TUP**	**COK**	
H1	1				1
H2	6	12	11	7	36
H3	7	9	15	11	42
H4	1	9			10
H5		4	3	1	8
H6		3			3
H7		3			3
H8		3			3
H9			11		11
H10			2		2
H11			2		2
H12				1	1
H13				1	1
**Total**	**15**	**43**	**44**	**21**	**123**

TGK: Tha Song Yang District, Tak Province (Greh-kee locality); TSL: Tha Song Yang District, Tak Province (Mae Salid Luang locality); TUP: Umphang District, Tak Province; COK: Omkoi District, Chiang Mai Province.

In contrast, haplotype H1 was observed only in Thailand, and haplotypes H9–H11 were newly identified and restricted to Thailand samples. Haplotype H8 was shared between Thailand and other Asian countries, including China, Laos and Myanmar.

### Neutrality tests

For the combined global dataset, neutrality tests indicated significant departures from mutation–drift equilibrium. Tajima’s D was negative and statistically significant (D = − 1.938, P < 0.05). Fu’s Fs was strongly negative (Fs = − 33.303). Fu and Li’s tests also yielded significantly negative values (D* = − 7.485, F* = − 5.718; P < 0.02).

In contrast, neutrality tests for the Thailand population alone were not statistically significant, including Tajima’s D = 0.259 (P > 0.10), Fu’s Fs = − 1.884, and Fu and Li’s D* = − 0.645 and F* = − 0.383.

### Phylogenetic analysis of the mitochondrial cox1 gene

Phylogenetic relationships were inferred using 344 aligned *A. lumbricoides cox1* sequences, including 123 newly generated sequences and 221 reference sequences (Supplementary Table 1). Three *Toxocara* species (*T. cati*, *T. canis*, and *T. malaysiensis*) were used as outgroups.

**Fig. 3:**
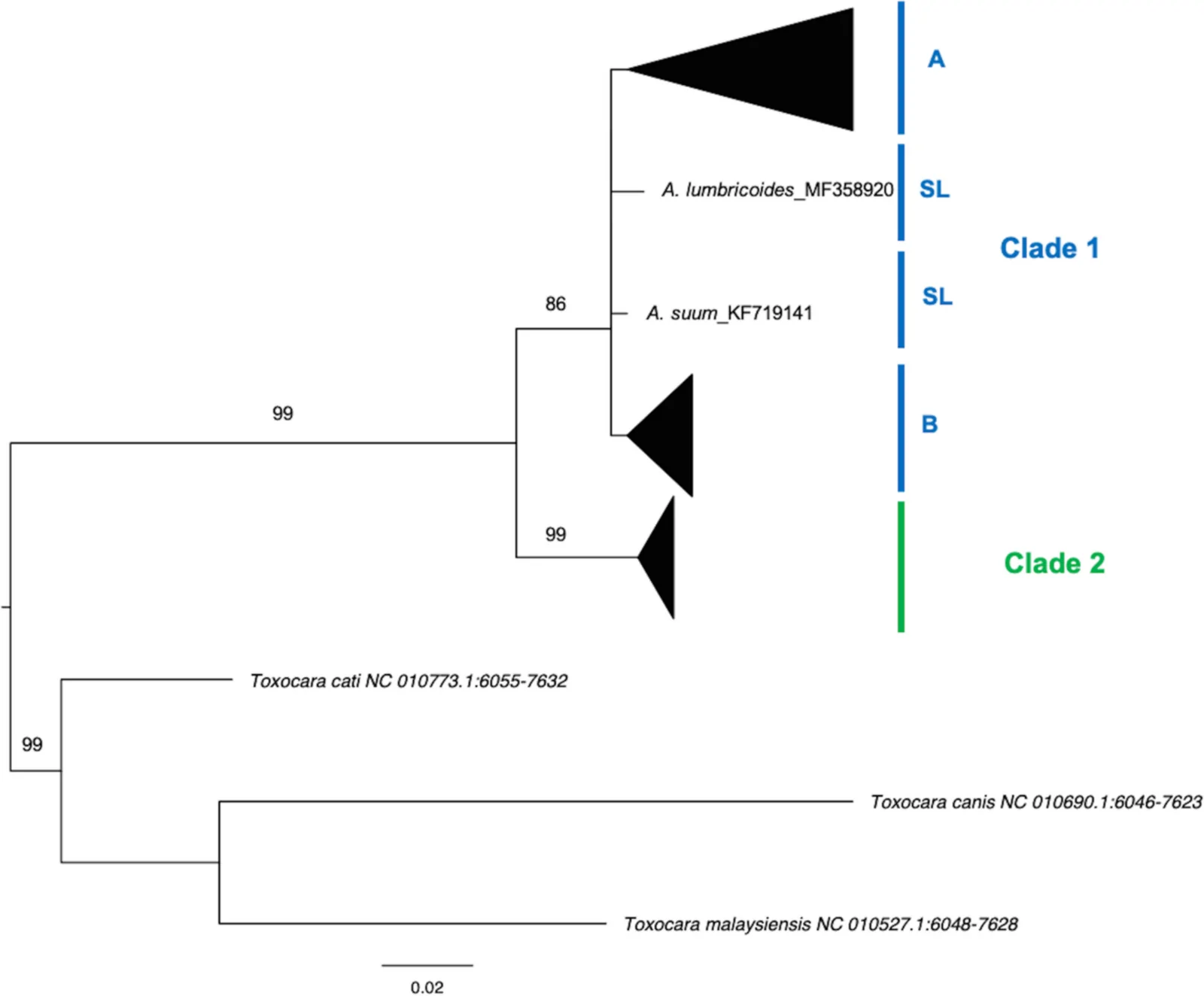
Maximum Likelihood phylogenetic tree based on partial mitochondrial *cox1* sequences. Maximum Likelihood tree inferred from 344 aligned *cox1* sequences (327 bp), including 123 sequences generated in this study and 221 reference sequences. The HKY + I model was selected based on BIC and applied in MEGA X. Bootstrap support was assessed with 1,000 replicates (values ≥ 50% shown). Two major clades were identified. Three *Toxocara* sequences were used as outgroups. Scale bar indicates substitutions per site

The best-fit substitution model (Hasegawa–Kishino–Yano model with invariant sites, HKY + I) was selected based on the Bayesian Information Criterion (BIC = 11491.82). Maximum likelihood (ML) analysis with 1,000 bootstrap replicates resolved two major clades (Clades 1 and 2) (Fig. 3). An overview of the phylogenetic relationships inferred from the *cox1* dataset is presented in Fig. 3, and the detailed tree topology is shown in Supplementary Fig. 6.

Clade 1 comprised the majority of sequences (n = 330) and exhibited a largely polytomous structure, consisting of subclade A (n = 270), subclade B (n = 58), and two singleton lineages. Notably, all sequences generated in this study (123/123, 100%) clustered within subclade A, representing 45.6% of the sequences in this subclade, whereas no Thai sequences were assigned to subclade B, the singleton lineages, or Clade 2. Subclade A was dominated by *A. lumbricoides*, although a small number of *A. suum* and unidentified *Ascaris* spp. sequences were also present.

Subclade B included primarily *A. lumbricoides*, along with *Ascaris* spp., *A. suum*, and *A. ovis*, including isolates from non-human hosts (chimpanzee, gibbon, and sheep).

Clade 2 (n = 14) was clearly distinct and comprised mainly *A. suum* and unidentified *Ascaris* spp., with no *A. lumbricoides* sequences detected. Outgroup sequences formed a well-supported basal lineage.

### Phylogenetic analysis and genetic relationships of the A. lumbricoides nad1 gene

**Fig. 4:**
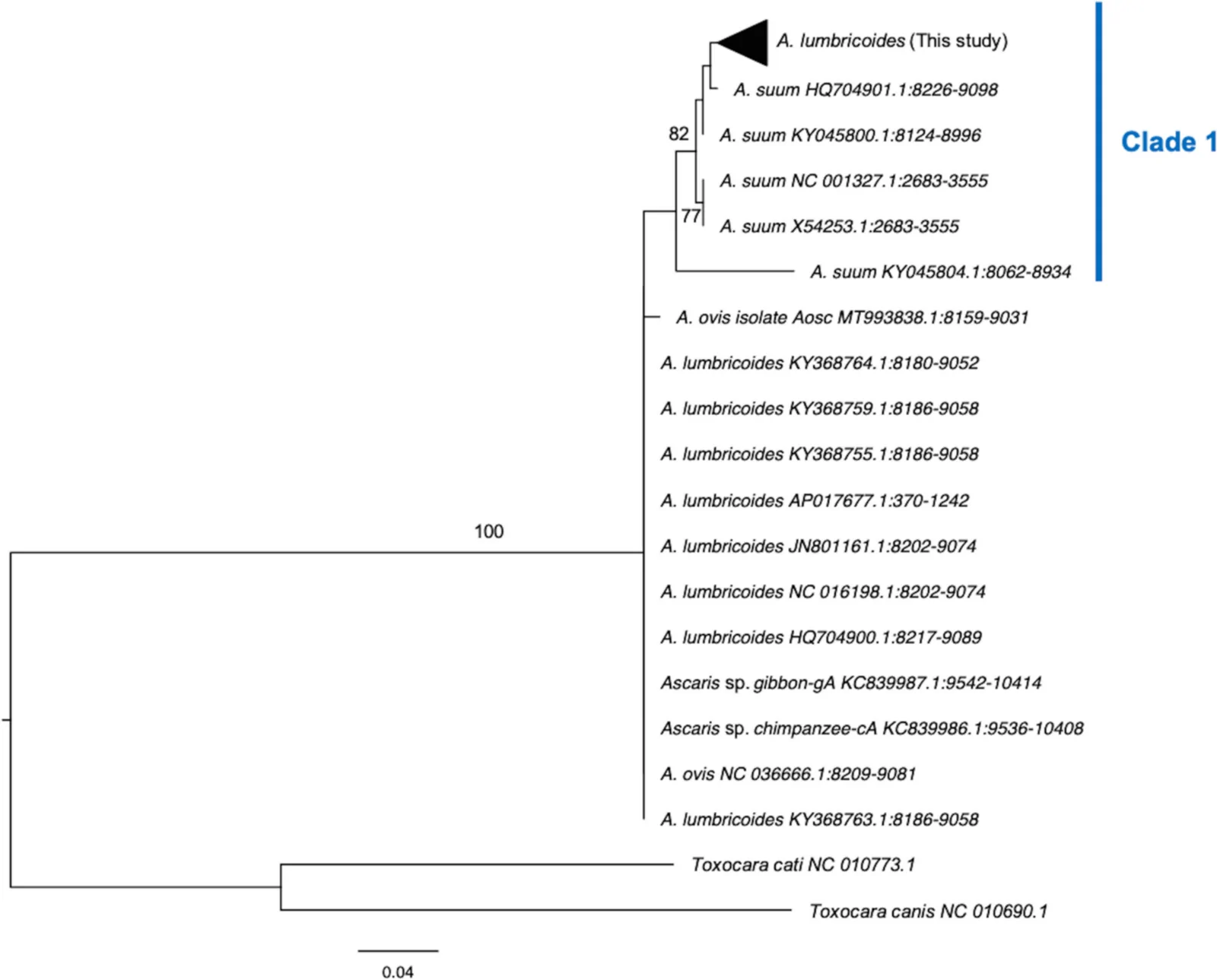
Maximum likelihood phylogenetic tree constructed from partial *nad1* sequences of *Ascaris* spp. The analysis included 137 sequences, comprising 120 newly generated sequences and 17 sequences obtained from public databases. Two *Toxocara* species were used as outgroups. The tree recovered a single major clade (Clade 1) comprising *A. lumbricoides* and *A. suum* sequences, with all sequences generated in this study clustering within Clade 1. Several samples formed a polytomy associated with Clade 1. Bootstrap support values (> 50%) are shown at the nodes

A total of 137 *nad1* sequences were analyzed, including 120 newly generated sequences and reference sequences from public databases. ML analysis under the HKY + G nucleotide substitution model recovered a major clade comprising *A. lumbricoides* and *A. suum* sequences (Fig. 4). The detailed tree topology is shown in Supplementary Fig. 7.

All sequences generated in this study clustered within clade 1. Some *A. suum* sequences grouped closely with *A. lumbricoides*, while one sequence (*A. suum* KY045804.1) showed greater divergence. A subset of sequences formed a polytomy, indicating unresolved relationships. Outgroup taxa were clearly separated with strong support.

### Phylogenetic analysis and genetic relationships based on concatenated A. lumbricoides cox1 and nad1 sequences

**Fig. 5:**
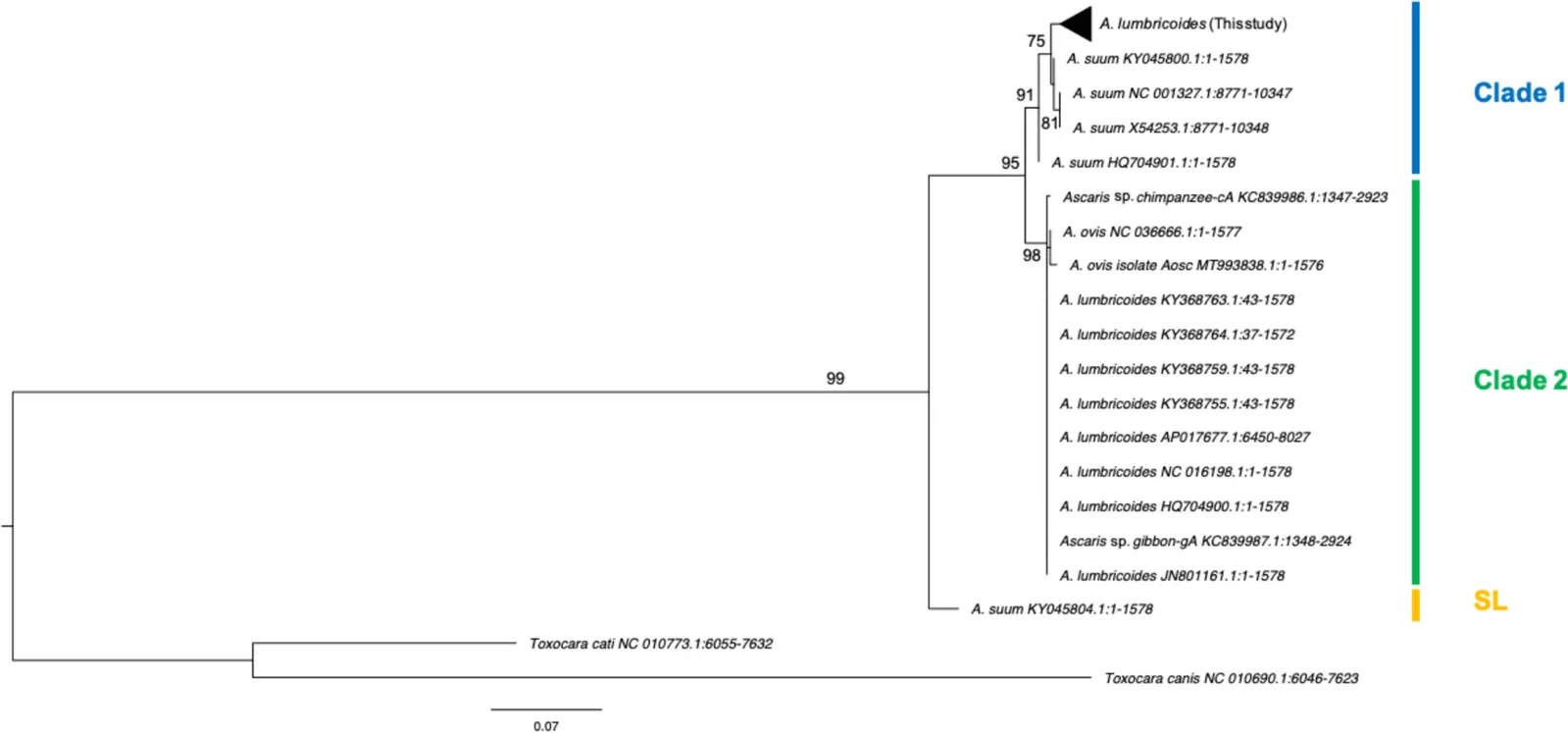
Maximum likelihood phylogenetic tree inferred from concatenated partial *cox1* and *nad1* sequences of *Ascaris* spp. The analysis included 123 sequences, comprising 106 newly generated concatenated sequences derived from adult worms, 17 *Ascaris* sequences retrieved from public databases, and two outgroup sequences (*T. canis* and *T. cati*)

Concatenated *cox1* and *nad1* sequences (n = 123) improved phylogenetic resolution compared to single-gene analyses. ML analysis under the HKY + G + I model recovered two well-supported clades (Fig. 5). The detailed composition of sequences within each clade is presented in Supplementary Fig. 8.

Clade 1 grouped *A. lumbricoides* with *A. suum*, suggesting close evolutionary relationships, whereas Clade 2 formed a distinct lineage comprising *A. lumbricoides*, *Ascaris* spp., and *A. ovis*.

The concatenated dataset reduced polytomy and resolved previously ambiguous relationships, indicating improved discriminatory power. Notably, one *A. suum* (KY045804) sequence formed a distinct lineage outside the two main clades, reflecting greater genetic divergence.

### Detection of benzimidazole resistance-associated mutations in the β-tubulin gene

**Fig. 6:**
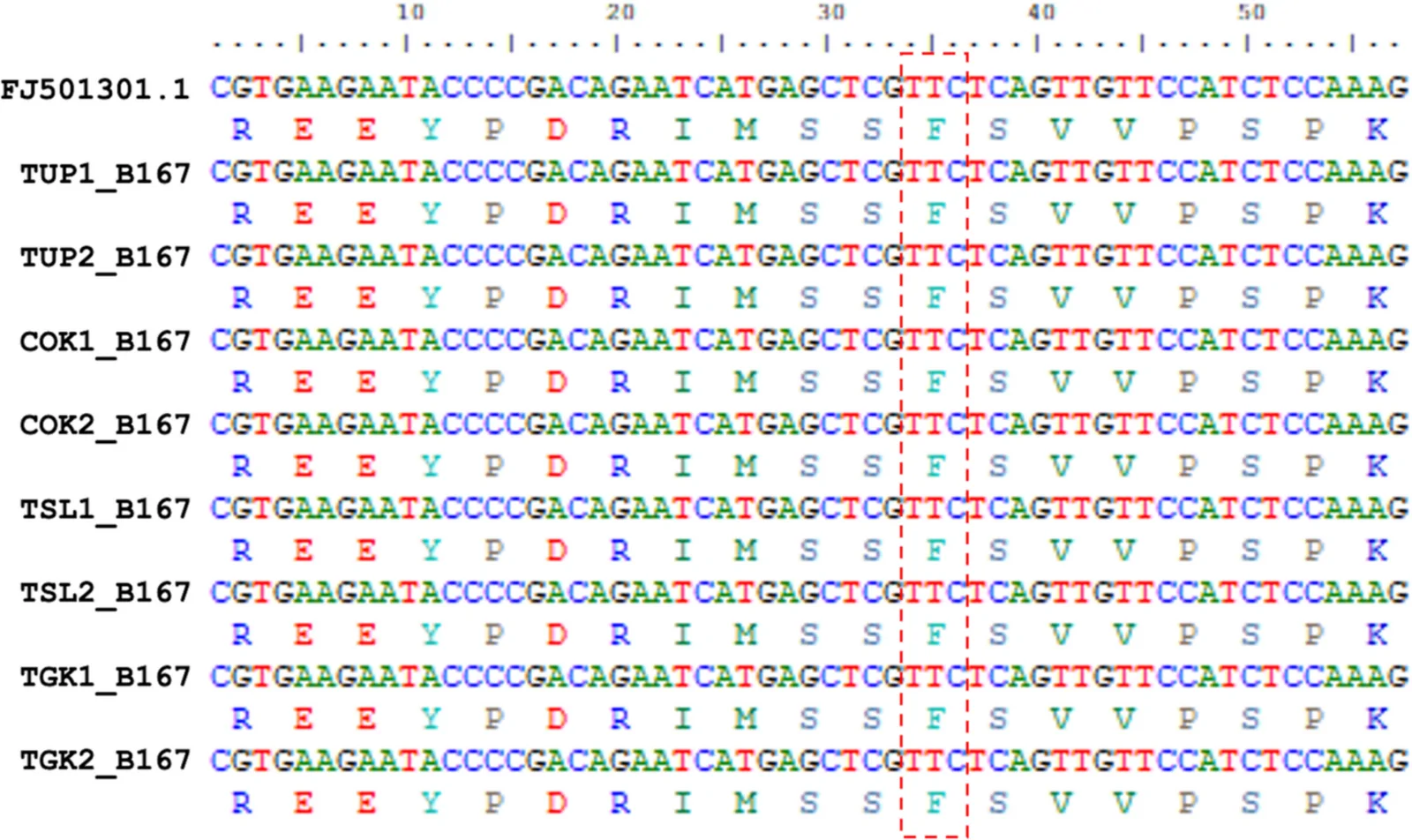
Representative *β-tubulin* sequences from adult *A. lumbricoides* show the multiple sequence alignment at codon 167, with the wild-type codon (TTC, encoding phenylalanine) indicated by a red box. TGK: Tha Song Yang District, Tak Province (Greh-kee locality); TSL: Tha Song Yang District, Tak Province (Mae Salid Luang locality); TUP: Umphang District, Tak Province; COK: Omkoi District, Chiang Mai Province

**Fig. 7:**
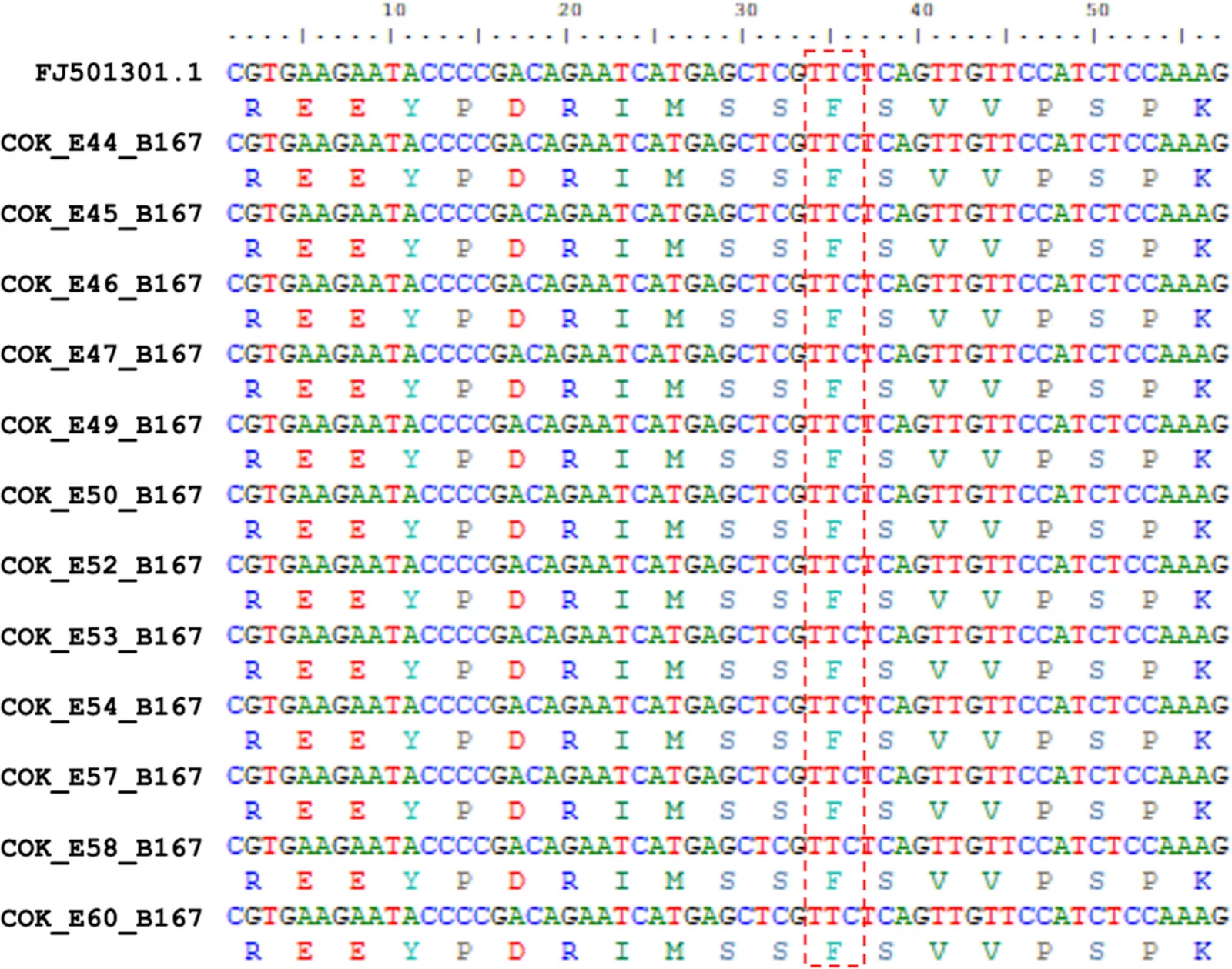
Representative *β-tubulin* sequences from *A. lumbricoides* individual eggs show the multiple sequence alignment at codon 167, with the wild-type codon (TTC, encoding Phenylalanine) indicated by a red box. COK_E: Omkoi District, Chiang Mai Province (gDNA was extracted from a single egg)

A total of 106 *A. lumbricoides β-tubulin* sequences were successfully analyzed. At codon 167, only the wild-type codon TTC (phenylalanine, F) was detected, with no evidence of the resistance-associated TAC (tyrosine, Y) mutation (Fig. 6). Additional analysis of individual eggs confirmed these findings, with all samples exhibiting the wild-type genotype (Fig. 7).

**Fig. 8:**
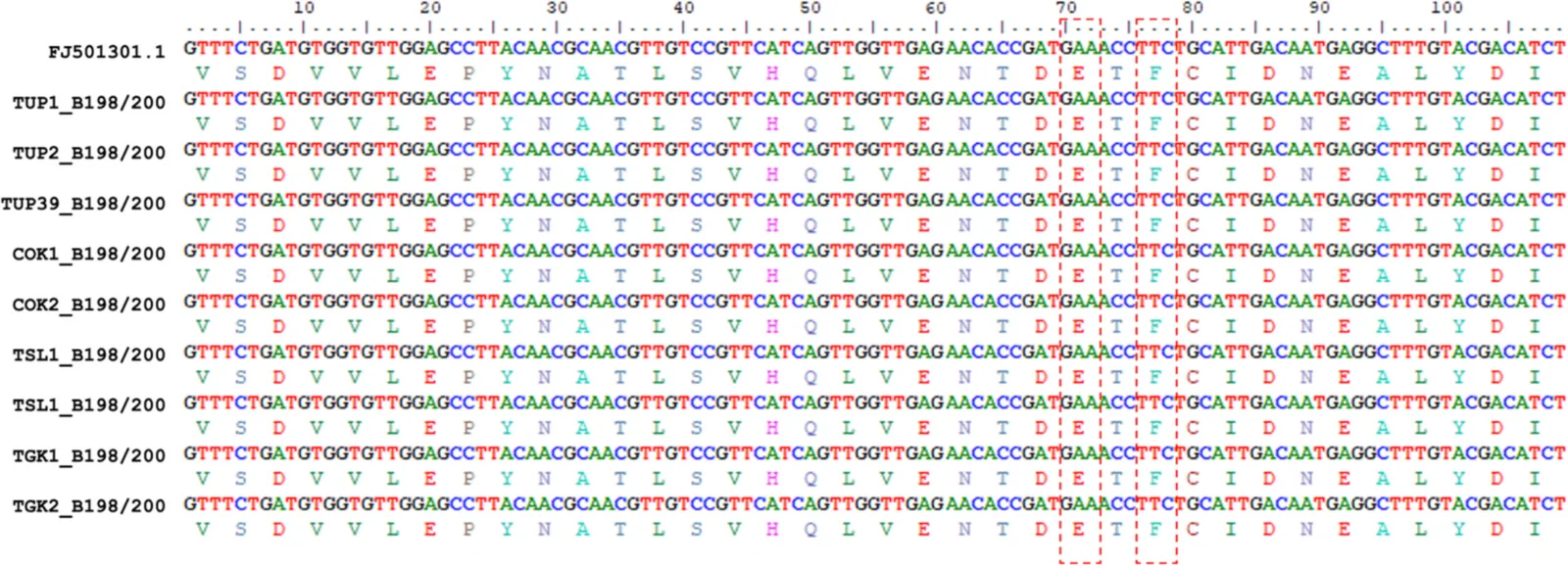
Representative *β-tubulin* sequences from adult *A. lumbricoides* show the multiple sequence alignment at codons 198 and 200, with the wild-type codons GAA (glutamic acid) and TTC (phenylalanine) indicated by a red box, respectively. TGK: Tha Song Yang District, Tak Province (Greh-kee locality); TSL: Tha Song Yang District, Tak Province (Mae Salid Luang locality); TUP: Umphang District, Tak Province; COK: Omkoi District, Chiang Mai Province

**Fig. 9:**
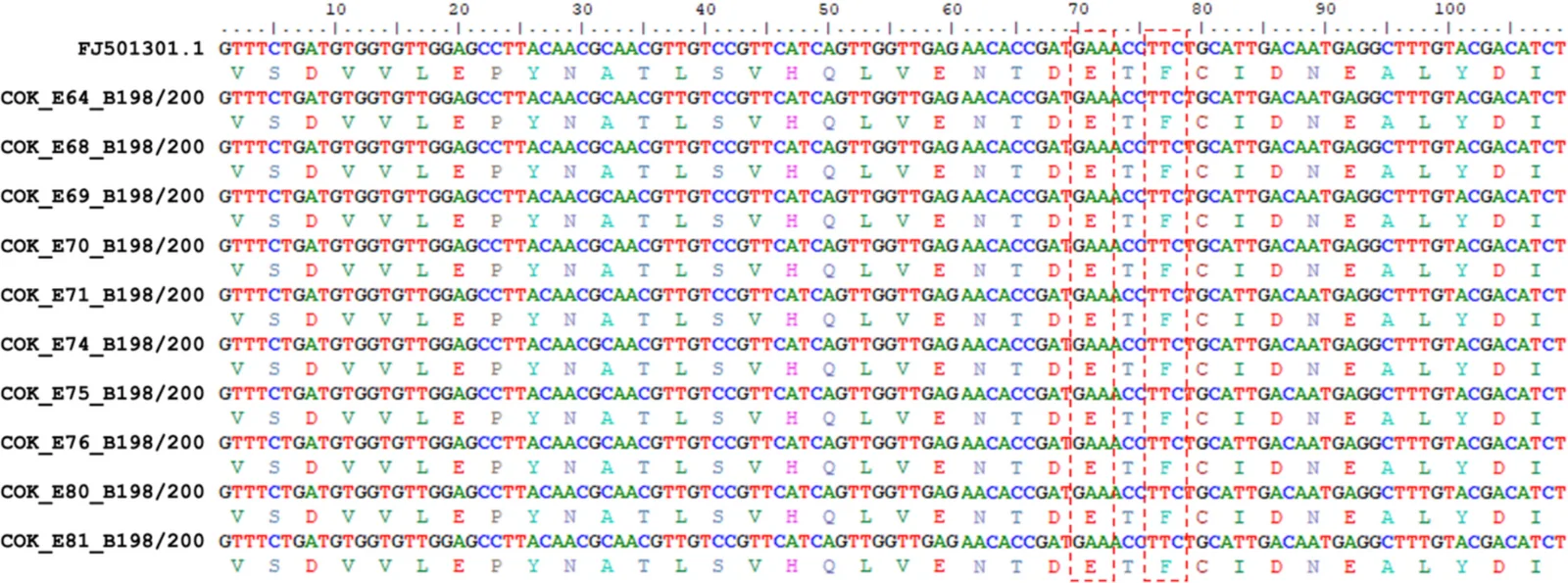
Representative *β-tubulin* sequences from *A. lumbricoides* individual eggs show the multiple sequence alignment at codons 198 and 200, with the wild-type codons GAA (glutamic acid) and TTC (phenylalanine) indicated by a red box, respectively. COK_E: Omkoi District, Chiang Mai Province (gDNA was extracted from a single egg)

Similarly, analysis of codons 198 and 200 revealed only the wild-type codons GAA (glutamic acid, E) and TTC (Fig. 8 and 9), with no detection of resistance-associated GCA (alanine, A) or TAC mutations, respectively.

## Discussion

The haplotype analysis identified 13 haplotypes within the Thailand dataset, including three novel haplotypes detected exclusively in this study, indicating ongoing genetic variation within the local population. Although the number of haplotypes identified locally was modest relative to the total sample size, the presence of newly observed variants suggests continued diversification of *A. lumbricoides* in this region. This pattern may reflect sustained local transmission dynamics and the movement of infected individuals between communities (Anderson 1995; Betson et al. 2014). In settings where pigs are commonly raised in close proximity to households, frequent human–pig contact may facilitate cross-transmission of *Ascaris* between hosts. Such interactions could contribute to haplotype mixing and help maintain or increase mitochondrial genetic diversity within endemic populations (Zhu et al 1999; Nejsum et al. 2005; Peng and Criscione 2012; Miller et al 2015; Sadaow et al. 2018; Monteiro et al. 2019; Easton et al. 2020).

Haplotype network analysis revealed three major clusters (A, B and C), consistent with clustering patterns reported previously (Sadaow et al. 2018). All *A. lumbricoides* sequences generated in the present study were assigned to Cluster A. Within this cluster, only a limited number of haplotypes were shared among multiple samples, whereas most haplotypes were represented by single sequences, reflecting substantial haplotype diversity.

Cluster A was the predominant lineage, comprising approximately 80% of the analyzed sequences and including isolates from multiple geographic regions. Its broad distribution and high haplotype diversity suggest that this lineage is widely established among *Ascaris* populations. Furthermore, the star-like network structure may reflect a history of demographic expansion from a limited number of ancestral lineages (Rogers and Harpending 1992; Avise 2000). The widespread occurrence of Cluster A across multiple geographic regions may have been facilitated by extensive dispersal and gene flow, potentially driven by long-term human migration and, particularly in endemic regions, by pig movement and human–pig transmission interfaces (Anderson 1995; Nejsum et al. 2012; Betson et al. 2014). Nevertheless, sampling bias cannot be excluded, as publicly available reference sequences are unevenly distributed across geographic regions and lineages, which may have contributed to the apparent predominance of Cluster A.

Cluster B comprised *Ascaris* sequences derived from a diverse range of host species, including humans, pigs, chimpanzees, gibbons and sheep, indicating limited host-specific segregation within this lineage. In contrast, Cluster C contained fewer haplotypes and was predominantly composed of pig-derived samples, suggesting a more restricted host association relative to Cluster B. Notably, the sequence *A. suum* (AB591803), which had previously been assigned to Cluster B, was positioned as a distinct lineage located between Clusters A and B in the present analysis. The intermediate placement of this sample may reflect genetic admixture, incomplete lineage sorting, or the accumulation of mutations that differentiate this sequence from typical Cluster B haplotypes, while changes in sampling composition and the inclusion of additional reference sequences are also known to alter haplotype network topology (Bandelt et al. 1999; Avise 2000; Posada and Crandall 2001; Criscione et al. 2005).

Phylogenetic analysis based on the *cox1* gene showed an overall clustering pattern consistent with the haplotype network. Sequences assigned to Clades 1A, 1B, and Clade 2 corresponded to Clusters A, B, and C, respectively, in the network analysis. All samples generated in the present study were grouped within the same major lineage in both approaches, indicating concordant genetic structuring at the clade level. However, two sequences (*A. suum* KF719141 and *A. lumbricoides* MF358920) did not cluster within Clade 1A (previously defined as Cluster A (Sadaow et al. 2018)) but instead formed distinct singleton lineages. Their inclusion resulted in a polytomy within Clade 1 of the *cox1* phylogeny, reflecting unresolved relationships among Clades 1A, 1B, and the singleton sequences. Such polytomies are commonly interpreted as indicative of limited phylogenetic resolution and may arise from insufficient informative sites, low sequence divergence within the analyzed region, or relatively recent divergence events. Moreover, expanded taxon sampling can introduce intermediate or divergent haplotypes that alter previously resolved topologies. The intermediate placement of KF719141 and MF358920 suggests a close evolutionary relationship with both Clades 1A and 1B; however, their precise positions cannot be confidently resolved using *cox1* data alone. Additional analyses incorporating other mitochondrial regions or nuclear markers would be necessary to further clarify their evolutionary relationships (Criscione et al. 2007; Liu et al. 2012; Zhou et al. 2012; Betson et al. 2014).

Although only a limited number of *Ascaris* sequences were available for the concatenated *cox1* and *nad1* analyses, the combined dataset yielded a more resolved phylogenetic topology compared with single-gene reconstructions. The *nad1*-only tree was characterized by a dominant clade with several polytomies, suggesting limited phylogenetic resolution, potentially due to insufficient informative sites and restricted sequence divergence within this marker. In contrast, concatenation of *cox1* and *nad1*, which are subject to different evolutionary constraints and substitution patterns, increased the number of informative characters and enhanced support for deeper nodes in the tree (Criscione et al. 2007; Liu et al. 2012). As a result, several relationships that were weakly supported or unresolved in the single-gene analyses showed improved resolution in the concatenated phylogeny. Notably, *A. suum* KY045804 was recovered as a distinct lineage, separated from Clades 1 and 2. The observed topological differences therefore most likely reflect improved phylogenetic signal derived from the combined dataset, underscoring the value of concatenated multilocus approaches for resolving relationships among closely related *Ascaris* lineages.

In the present study, haplotype network and phylogenetic analyses based on the *cox1* and *nad1* genes did not resolve *A. lumbricoides* samples into geographically distinct populations. All samples from the three study regions clustered within a previously defined clade (Sadaow et al. 2018) that comprises both human- and pig-derived *Ascaris* isolates from multiple geographic areas, without a clear spatial structure. The absence of species- or geography-specific clustering is consistent with earlier reports and reflects the documented genetic overlap between *A. lumbricoides* and *A. suum*. Molecular investigations using mitochondrial and nuclear markers have shown low levels of genetic divergence between these taxa, supporting the view that they may represent a single species or very recently diverged host-associated lineages (Anderson et al. 1993; Leles et al. 2012; Betson et al. 2014). As a result, mitochondrial haplotypes are often shared between worms infecting humans and pigs, limiting species-level resolution in phylogenetic analyses based solely on mitochondrial data.

The present findings raise the possibility that a proportion of human infections may result from direct zoonotic transmission of porcine-derived *Ascaris*, including *A. suum*. In endemic settings where humans and pigs coexist in close proximity, particularly under conditions of inadequate sanitation, humans may be directly exposed to infective eggs originating from pig fecal contamination in the environment. This provides a plausible transmission pathway for human ascariasis involving porcine *Ascaris* populations (Nejsum et al. 2005, 2012; Betson et al. 2014). Therefore, the observed genetic patterns may reflect ongoing zoonotic transmission driven by direct environmental exposure at the human–pig interface.

Overall, the clustering of human- and pig-derived *Ascaris* samples within a single genetic lineage, together with the absence of clear geographic structuring, aligns with current understanding of *Ascaris* population genetics and is compatible with the existence of overlapping transmission cycles between humans and pigs. However, these findings also highlight the limitations of relying solely on mitochondrial markers to resolve species boundaries or fine-scale population structure. Future investigations incorporating multiple nuclear loci or genome-wide data, combined with detailed host and epidemiological information, will be necessary to more accurately elucidate the evolutionary relationships and transmission dynamics of *Ascaris* populations.

In this study, analysis of the *β-tubulin* gene at codons 167, 198, and 200 revealed only wild-type alleles in all *A. lumbricoides* samples examined. No single nucleotide polymorphisms corresponding to the amino acid substitutions at codons 167, 198, or 200 were detected. These results indicate an absence of molecular evidence for benzimidazole resistance in the *A. lumbricoides* population investigated. Although, previous study has reported for the mutations at codon 167 in samples from Haiti, Kenya, and Panama (Diawara et al. 2009), and the mutation at codon 200 in Brazil (Furtado et al. 2019). In contrast, several studies reported the absence of resistance-associated mutations at codons 167, 198, and 200 in *A. lumbricoides* (Diawara et al. 2009, 2013; Sutherland and Leathwick 2011; Krücken et al. 2017; Rashwan et al. 2017; Jones et al. 2022). However, these same mutations are widely documented and strongly associated with benzimidazole resistance in intestinal trichostrongylid nematodes of livestock, particularly *H. contortus* (Wolstenholme et al. 2004; Ghisi et al. 2007; Sutherland and Leathwick 2011). The apparently low frequency of *β-tubulin* mutations in *A. lumbricoides* suggests that the development of anthelmintic resistance in this genus may differ from that observed in *H. contortus*. This difference may be partly explained by lower treatment frequency in human populations compared with the intensive and repeated use of anthelmintics in livestock, such as sheep and goats, which results in greater drug selection pressure. Increased drug pressure is known to accelerate the selection of resistant nematode strains in livestock. Consequently, *Ascaris* populations may be subject to different selective pressures, potentially limiting the fixation and spread of resistance-associated alleles (Gilleard and Beech 2007; Vercruysse et al. 2011). Another possible explanation for the absence of detected mutations is that resistant alleles, if present, occur at very low frequencies and may not be detected due to limited sample size or the number of eggs analyzed per infection. Although a mutation was detected at codon 167, this mutation has not been shown to affect drug efficacy in *A. lumbricoides* (Sutherland and Leathwick 2011).

Although mutations at *β-tubulin* codons 167, 198, and 200 are the primary molecular markers associated with benzimidazole resistance, alternative resistance mechanisms have also been proposed in nematodes, including enhanced drug efflux mediated by P-glycoproteins and metabolic detoxification involving cytochrome P450 enzymes (Kotze and Prichard 2016; Vosála et al. 2025). Such mechanisms would not be detected by the targeted analysis of single nucleotide polymorphisms performed in the present study. Therefore, while no evidence of resistance-associated *β-tubulin* mutations was identified, the existence of alternative mechanisms contributing to reduced drug susceptibility cannot be completely excluded. Further studies incorporating functional assays and broader genomic approaches are needed to investigate the potential contribution of non-target-site resistance mechanisms to benzimidazole susceptibility in *Ascaris* populations.

The present findings provide no molecular evidence of benzimidazole resistance in the *A. lumbricoides* population examined. Nevertheless, continued monitoring is recommended, particularly in settings where mass drug administration programs are ongoing or expanding. Integrating molecular surveillance with phenotypic drug efficacy assessments will be important for the early detection of emerging resistance (Gilleard and Beech 2007; Diawara et al. 2009).

A technical limitation of the present study was the occasional failure of PCR amplification from individual eggs. Although extensive washing and purification steps were performed following egg recovery to minimize the carryover of fecal material and associated PCR inhibitors, trace contaminants cannot be completely excluded. In addition, the highly resistant nature of the *Ascaris* eggshell may have resulted in incomplete mechanical disruption of some eggs, thereby limiting DNA release and reducing PCR amplification efficiency. Consequently, the absence of amplification in some individual eggs should be interpreted with caution and does not necessarily indicate the absence of target DNA.

## Conclusions

This study demonstrates measurable genetic diversity in *A. lumbricoides* populations from Thailand, including the identification of three novel haplotypes. Although haplotype diversity at the local scale was lower than that observed globally, the presence of multiple haplotypes indicates ongoing genetic variation within endemic populations. The absence of clear geographic structuring is consistent with ongoing gene flow and population connectivity among regions.

The observed genetic overlap with *A. suum* supports a shared evolutionary background and underscores the potential contribution of zoonotic transmission. No molecular evidence of benzimidazole resistance was identified at the examined *β-tubulin* codons.

Overall, these findings provide baseline molecular data and emphasize the importance of continued surveillance using expanded sampling and multilocus or genome-wide approaches to further clarify transmission dynamics and monitor the potential emergence of anthelmintic resistance.

## Supplementary Information

Below is the link to the electronic supplementary material.

Supplementary file1 (DOCX 15308 KB)

## Data Availability

All data generated or analyzed during this study are included in this article and its supplementary information files. The sequence data have been deposited in GenBank (NCBI) and are accessible under the accession numbers provided in Supplementary Table 1.
