# Supplementary material for: Genetic diversity, population structure, and benzimidazole resistance profiling of Ascaris lumbricoides in northwestern Thailand
Source: Parasitol Res. 2026 Jul 20;125(1):124. doi: 10.1007/s00436-026-08727-w (PMC13669075; doi:10.1007/s00436-026-08727-w)
Supplement: Supplementary file 1 [file 436_2026_8727_MOESM1_ESM.docx]

**Supplementary Figure 1** Enlarged views of the sampling locations shown in Fig. 1: (A) Tha Song Yang District, Tak Province; (B) Umphang District, Tak Province; and (C) Omkoi District, Chiang Mai Province. Detailed maps were generated using Google My Maps with Google Earth satellite imagery as the basemap.

 **A**

 **B**

 **C**

**Supplementary Figure 2** Agarose gel electrophoresis of PCR products of mitochondrial *cox1* gene from *A. lumbricoides* (~431 bp). Lane M: 1 kb DNA ladder (GeneRuler 1 kb Plus, Thermo Fisher Scientific Inc.). Lanes 1–6: PCR products from six individual samples. Lane 7: non-template control (NTC).

**Supplementary Figure 3** Agarose gel electrophoresis of PCR products of mitochondrial *nad1* gene from *A. lumbricoides* (~400 bp). Lane M: 1 kb DNA ladder (GeneRuler 1 kb Plus, Thermo Fisher Scientific Inc.). Lanes 1–6: PCR products from six individual samples. Lane 7: non-template control (NTC).

**Supplementary Figure 4** Agarose gel electrophoresis of PCR products generated from (A) first-round amplification of *β-tubulin* at codon 167 (~398 bp) and (B) second-round amplification at codon 167 (~129 bp). Lane M: 1 kb DNA ladder (GeneRuler 1 kb Plus, Thermo Fisher Scientific Inc.). Lanes 1–6: PCR products from six individual samples.

**Supplementary Figure 5** Agarose gel electrophoresis of PCR products generated from (A) first-round amplification of *β-tubulin* 198 and 200 (~191 bp) and (B) second-round amplification at codon 198 and 200 (~179bp). Lane M: 1 kb DNA ladder (GeneRuler 1 kb Plus, Thermo Fisher Scientific Inc.). Lanes 1–6: PCR products from six individual samples.

**Supplementary Figure 6** Detailed composition of *cox1* sequences assigned to clade 1 and clade 2 in the phylogenetic analysis, showing the distribution of newly generated and previously reported sequences within subclades A and B, as well as two independent singleton lineages. Bootstrap values (>50%) are shown at the nodes. Scale bar represents substitutions per site. TGK: Tha Song Yang District, Tak Province (Greh-kee locality); TSL: Tha Song Yang District, Tak Province (Salid Luang locality); TUP: Umphang District, Tak Province; COK: Omkoi District, Chiang Mai Province; COK_E: Omkoi District, Chiang Mai Province (genomic DNA was extracted from a single egg).

**Supplementary Figure 7** Detailed composition of *nad1* sequences assigned to clade 1 and the associated polytomous samples identified in the phylogenetic analysis. Bootstrap values (>50%) are shown at the nodes. TGK: Tha Song Yang District, Tak Province (Greh-kee locality); TSL: Tha Song Yang District, Tak Province (Salid Luang locality); TUP: Umphang District, Tak Province; COK: Omkoi District, Chiang Mai Province; COK_E: Omkoi District, Chiang Mai Province (genomic DNA was extracted from a single egg).

**Supplementary Figure 8** Detailed composition of *Ascaris* sequences assigned to clade 1, clade 2, and a singleton lineage based on the concatenated *cox1–nad1* phylogenetic analysis. Bootstrap values (>50%) are shown at the nodes. TGK: Tha Song Yang District, Tak Province (Greh-kee locality); TSL: Tha Song Yang District, Tak Province (Salid Luang locality); TUP: Umphang District, Tak Province; COK: Omkoi District, Chiang Mai Province; COK_E: Omkoi District, Chiang Mai Province (genomic DNA was extracted from a single egg).

**Supplementary Table 1** Sample information, haplotype assignment, cluster classification, and accession numbers of *Ascaris* isolates included in this study.

| **No.** | **Sample ID** | **Species identification** | **cox1 GenBank**  **Accession No.** | **nad1 GenBank**  **Accession No.** | **β-tubulin Codon 167 GenBank**  **Accession No.** | **β-tubulin Codon 198/200 GenBank**  **Accession No.** | **Haplotype** | **Cluster** |
| --- | --- | --- | --- | --- | --- | --- | --- | --- |
| 1 | AB591795 | *A. lumbricoides* | AB591795 | NA | NA | NA | 24 | B |
| 2 | AB591796 | *A. lumbricoides* | AB591796 | NA | NA | NA | 38 | B |
| 3 | AB591797 | *A. lumbricoides* | AB591797 | NA | NA | NA | 38 | B |
| 4 | AB591798 | *A. lumbricoides* | AB591798 | NA | NA | NA | 38 | B |
| 5 | AB591799 | *A. lumbricoides* | AB591799 | NA | NA | NA | 38 | B |
| 6 | AB591800 | *A. lumbricoides* | AB591800 | NA | NA | NA | 38 | B |
| 7 | AB591801 | *A. lumbricoides* | AB591801 | NA | NA | NA | 90 | A |
| 8 | AB591802 | *A. suum* | AB591802 | NA | NA | NA | 51 | A |
| 9 | AB591803 | *A. suum* | AB591803 | NA | NA | NA | 39 | SL |
| 10 | AB591804 | *A. suum* | AB591804 | NA | NA | NA | 38 | B |
| 11 | AB591805 | *A. suum* | AB591805 | NA | NA | NA | 38 | B |
| 12 | AJ968324 | *A. lumbricoides* | AJ968324 | NA | NA | NA | 56 | A |
| 13 | AJ968325 | *A. lumbricoides* | AJ968325 | NA | NA | NA | 80 | A |
| 14 | AJ968326 | *A. lumbricoides* | AJ968326 | NA | NA | NA | 71 | A |
| 15 | AJ968327 | *A. lumbricoides* | AJ968327 | NA | NA | NA | 55 | A |
| 16 | AJ968328 | *A. lumbricoides* | AJ968328 | NA | NA | NA | 59 | A |
| 17 | AJ968329 | *A. lumbricoides* | AJ968329 | NA | NA | NA | 57 | A |
| 18 | AJ968330 | *A. lumbricoides* | AJ968330 | NA | NA | NA | 25 | B |
| 19 | AJ968332 | *A. lumbricoides* | AJ968332 | NA | NA | NA | 38 | B |
| 20 | AJ968333 | *A. lumbricoides* | AJ968333 | NA | NA | NA | 8 | A |
| 21 | AJ968335 | *A. suum* | AJ968335 | NA | NA | NA | 60 | A |
| 22 | AJ968336 | *A. suum* | AJ968336 | NA | NA | NA | 2 | A |
| 23 | AJ968337 | *A. suum* | AJ968337 | NA | NA | NA | 23 | B |
| 24 | AJ968338 | *A. suum* | AJ968338 | NA | NA | NA | 38 | B |
| 25 | AJ968339 | *A. suum* | AJ968339 | NA | NA | NA | 22 | B |
| 26 | AJ968340 | *A. suum* | AJ968340 | NA | NA | NA | 37 | B |
| 27 | AJ968341 | *A. suum* | AJ968341 | NA | NA | NA | 35 | B |
| 28 | AJ968343 | *A. suum* | AJ968343 | NA | NA | NA | 21 | B |
| 29 | AP017677.1:6450-8027 | *A. lumbricoides* | AP017677.1:6450-8027 | AP017677.1:6450-8027 | NA | NA | 38 | B |
| 30 | EU582484 | *A. lumbricoides* | EU582484 | NA | NA | NA | 2 | A |
| 31 | EU582485 | *A. lumbricoides* | EU582485 | NA | NA | NA | 12 | A |
| 32 | EU582486 | *A. lumbricoides* | EU582486 | NA | NA | NA | 32 | B |
| 33 | EU582487 | *A. lumbricoides* | EU582487 | NA | NA | NA | 66 | A |
| 34 | EU582488 | *A. lumbricoides* | EU582488 | NA | NA | NA | 78 | A |
| 35 | EU582489 | *A. lumbricoides* | EU582489 | NA | NA | NA | 64 | A |
| 36 | EU582490 | *A. lumbricoides* | EU582490 | NA | NA | NA | 38 | B |
| 37 | EU582491 | *A. lumbricoides* | EU582491 | NA | NA | NA | 3 | A |
| 38 | EU582492 | *A. lumbricoides* | EU582492 | NA | NA | NA | 2 | A |
| 39 | EU582494 | *A. lumbricoides* | EU582494 | NA | NA | NA | 13 | A |
| 40 | EU582495 | *A. lumbricoides* | EU582495 | NA | NA | NA | 75 | A |
| 41 | EU582496 | *A. lumbricoides* | EU582496 | NA | NA | NA | 74 | A |
| 42 | EU582497 | *A. lumbricoides* | EU582497 | NA | NA | NA | 2 | A |
| 43 | EU582498 | *A. lumbricoides* | EU582498 | NA | NA | NA | 73 | A |
| 44 | EU582499 | *A. lumbricoides* | EU582499 | NA | NA | NA | 31 | B |
| 45 | EU628687.1 | *Ascaris* sp*.* Pt TW-2008 | EU628687.1 | NA | NA | NA | 23 | B |
| 46 | EU628688.1 | *Ascaris* sp. H TW-2008 | EU628688.1 | NA | NA | NA | 38 | B |
| 47 | GU326948 | *A. lumbricoides* | GU326948 | NA | NA | NA | 2 | A |
| 48 | GU326949 | *A. lumbricoides* | GU326949 | NA | NA | NA | 2 | A |
| 49 | GU326950 | *A. lumbricoides* | GU326950 | NA | NA | NA | 2 | A |
| 50 | GU326951 | *A. suum* | GU326951 | NA | NA | NA | 2 | A |
| 51 | GU326952 | *A. lumbricoides* | GU326952 | NA | NA | NA | 38 | B |
| 52 | GU326953 | *A. lumbricoides* | GU326953 | NA | NA | NA | 30 | B |
| 53 | GU326954 | *A. lumbricoides* | GU326954 | NA | NA | NA | 93 | A |
| 54 | GU326955 | *A. lumbricoides* | GU326955 | NA | NA | NA | 61 | A |
| 55 | HM602025 | *A. suum* | HM602025 | NA | NA | NA | 4 | A |
| 56 | HQ704900.1:1-1578 | *A. lumbricoides* | HQ704900.1:1-1578 | HQ704900.1:1-1578 | NA | NA | 38 | B |
| 57 | HQ704901.1:1-1578 | *A. suum* | HQ704901.1:1-1578 | HQ704901.1:1-1578 | NA | NA | 105 | A |
| 58 | JN575625 | *Ascaris* sp. | JN575625 | NA | NA | NA | 38 | B |
| 59 | JN575626 | *Ascaris* sp. | JN575626 | NA | NA | NA | 38 | B |
| 60 | JN575627 | *Ascaris* sp. | JN575627 | NA | NA | NA | 38 | B |
| 61 | JN575628 | *Ascaris* sp. | JN575628 | NA | NA | NA | 38 | B |
| 62 | JN575629 | *Ascaris* sp. | JN575629 | NA | NA | NA | 5 | A |
| 63 | JN575630 | *Ascaris* sp. | JN575630 | NA | NA | NA | 57 | A |
| 64 | JN575631 | *Ascaris* sp. | JN575631 | NA | NA | NA | 38 | B |
| 65 | JN575632 | *Ascaris* sp. | JN575632 | NA | NA | NA | 16 | C |
| 66 | JN575633 | *Ascaris* sp. | JN575633 | NA | NA | NA | 15 | C |
| 67 | JN801161.1:1-1578 | *A. lumbricoides* | JN801161.1:1-1578 | JN801161.1:1-1578 | NA | NA | 38 | B |
| 68 | KC455923.1 | *Ascaris* sp. | KC455923.1 | NA | NA | NA | 16 | C |
| 69 | KC455924 | *Ascaris* sp. | KC455924 | NA | NA | NA | 17 | C |
| 70 | KC455925 | *Ascaris* sp. | KC455925 | NA | NA | NA | 15 | C |
| 71 | KC455926 | *Ascaris* sp. | KC455926 | NA | NA | NA | 14 | C |
| 72 | KC455927.1 | *Ascaris* sp. | KC455927.1 | NA | NA | NA | 5 | A |
| 73 | KC455928 | *Ascaris* sp. | KC455928 | NA | NA | NA | 48 | A |
| 74 | KC455929 | *Ascaris* sp. | KC455929 | NA | NA | NA | 38 | B |
| 75 | KC455930 | *Ascaris* sp. | KC455930 | NA | NA | NA | 50 | A |
| 76 | KC455931 | *Ascaris* sp. | KC455931 | NA | NA | NA | 49 | A |
| 77 | KC455932 | *Ascaris* sp. | KC455932 | NA | NA | NA | 47 | A |
| 78 | KC455934.1 | *Ascaris* sp. | KC455934.1 | NA | NA | NA | 2 | A |
| 79 | KC455935 | *Ascaris* sp. | KC455935 | NA | NA | NA | 28 | B |
| 80 | KC839986.1 | *Ascaris* sp. chimpanzee-cA | KC839986.1 | KC839986.1 | NA | NA | 23 | B |
| 81 | KC839987.1 | *Ascaris* sp. gibbon-gA | KC839987.1 | KC839987.1 | NA | NA | 38 | B |
| 82 | KF536859 | *A. lumbricoides* | KF536859 | NA | NA | NA | 72 | A |
| 83 | KF536860 | *A. lumbricoides* | KF536860 | NA | NA | NA | 69 | A |
| 84 | KF536861 | *A. lumbricoides* | KF536861 | NA | NA | NA | 70 | A |
| 85 | KF536862 | *A. lumbricoides* | KF536862 | NA | NA | NA | 76 | A |
| 86 | KF536863 | *A. lumbricoides* | KF536863 | NA | NA | NA | 83 | A |
| 87 | KF536864 | *A. lumbricoides* | KF536864 | NA | NA | NA | 77 | A |
| 88 | KF536865 | *A. lumbricoides* | KF536865 | NA | NA | NA | 2 | A |
| 89 | KF536866 | *A. lumbricoides* | KF536866 | NA | NA | NA | 7 | A |
| 90 | KF536867 | *A. lumbricoides* | KF536867 | NA | NA | NA | 62 | A |
| 91 | KF536869 | *A. lumbricoides* | KF536869 | NA | NA | NA | 6 | A |
| 92 | KF536870 | *A. lumbricoides* | KF536870 | NA | NA | NA | 5 | A |
| 93 | KF536871 | *A. lumbricoides* | KF536871 | NA | NA | NA | 32 | B |
| 94 | KF536872 | *A. lumbricoides* | KF536872 | NA | NA | NA | 33 | B |
| 95 | KF719094 | *A. lumbricoides* | KF719094 | NA | NA | NA | 95 | A |
| 96 | KF719095 | *Ascaris* sp. | KF719095 | NA | NA | NA | 4 | A |
| 97 | KF719096 | *Ascaris* sp. | KF719096 | NA | NA | NA | 4 | A |
| 98 | KF719097 | *Ascaris* sp. | KF719097 | NA | NA | NA | 4 | A |
| 99 | KF719099 | *Ascaris* sp. | KF719099 | NA | NA | NA | 4 | A |
| 100 | KF719100 | *Ascaris* sp. | KF719100 | NA | NA | NA | 2 | A |
| 101 | KF719101 | *Ascaris* sp. | KF719101 | NA | NA | NA | 2 | A |
| 102 | KF719102 | *A. lumbricoides* | KF719102 | NA | NA | NA | 81 | A |
| 103 | KF719103 | *A. lumbricoides* | KF719103 | NA | NA | NA | 76 | A |
| 104 | KF719104 | *A. lumbricoides* | KF719104 | NA | NA | NA | 2 | A |
| 105 | KF719105 | *A. lumbricoides* | KF719105 | NA | NA | NA | 65 | A |
| 106 | KF719106 | *A. lumbricoides* | KF719106 | NA | NA | NA | 82 | A |
| 107 | KF719107 | *A. lumbricoides* | KF719107 | NA | NA | NA | 86 | A |
| 108 | KF719108 | *A. lumbricoides* | KF719108 | NA | NA | NA | 75 | A |
| 109 | KF719109 | *A. lumbricoides* | KF719109 | NA | NA | NA | 26 | B |
| 110 | KF719110 | *A. lumbricoides* | KF719110 | NA | NA | NA | 88 | A |
| 111 | KF719111 | *A. lumbricoides* | KF719111 | NA | NA | NA | 92 | A |
| 112 | KF719112 | *A. lumbricoides* | KF719112 | NA | NA | NA | 4 | A |
| 113 | KF719113 | *A. lumbricoides* | KF719113 | NA | NA | NA | 84 | A |
| 114 | KF719114 | *A. lumbricoides* | KF719114 | NA | NA | NA | 79 | A |
| 115 | KF719115 | *A. lumbricoides* | KF719115 | NA | NA | NA | 85 | A |
| 116 | KF719116 | *A. lumbricoides* | KF719116 | NA | NA | NA | 54 | A |
| 117 | KF719117 | *A. lumbricoides* | KF719117 | NA | NA | NA | 63 | A |
| 118 | KF719118 | *A. lumbricoides* | KF719118 | NA | NA | NA | 34 | B |
| 119 | KF719119 | *A. lumbricoides* | KF719119 | NA | NA | NA | 91 | A |
| 120 | KF719120 | *Ascaris* sp. | KF719120 | NA | NA | NA | 5 | A |
| 121 | KF719121 | *Ascaris* sp. | KF719121 | NA | NA | NA | 5 | A |
| 122 | KF719122 | *A. suum* | KF719122 | NA | NA | NA | 5 | A |
| 123 | KF719123 | *A. suum* | KF719123 | NA | NA | NA | 27 | B |
| 124 | KF719124 | *A. suum* | KF719124 | NA | NA | NA | 36 | B |
| 125 | KF719125 | *A. suum* | KF719125 | NA | NA | NA | 53 | A |
| 126 | KF719126 | *A. suum* | KF719126 | NA | NA | NA | 52 | A |
| 127 | KF719127 | *A. suum* | KF719127 | NA | NA | NA | 19 | C |
| 128 | KF719128 | *A. suum* | KF719128 | NA | NA | NA | 87 | A |
| 129 | KF719129 | *A. suum* | KF719129 | NA | NA | NA | 5 | A |
| 130 | KF719130 | *A. suum* | KF719130 | NA | NA | NA | 68 | A |
| 131 | KF719131 | *A. suum* | KF719131 | NA | NA | NA | 16 | C |
| 132 | KF719132 | *A. suum* | KF719132 | NA | NA | NA | 16 | C |
| 133 | KF719133 | *A. suum* | KF719133 | NA | NA | NA | 16 | C |
| 134 | KF719134 | *A. suum* | KF719134 | NA | NA | NA | 16 | C |
| 135 | KF719135 | *A. suum* | KF719135 | NA | NA | NA | 67 | A |
| 136 | KF719136 | *A. suum* | KF719136 | NA | NA | NA | 58 | A |
| 137 | KF719137 | *A. suum* | KF719137 | NA | NA | NA | 58 | A |
| 138 | KF719138 | *A. suum* | KF719138 | NA | NA | NA | 29 | B |
| 139 | KF719139 | *Ascaris* sp. | KF719139 | NA | NA | NA | 40 | A |
| 140 | KF719140 | *Ascaris* sp. | KF719140 | NA | NA | NA | 40 | A |
| 141 | KF719141 | *A. suum* | KF719141 | NA | NA | NA | 96 | A |
| 142 | KF719142 | *A. suum* | KF719142 | NA | NA | NA | 20 | C |
| 143 | KF719143 | *Ascaris* sp. | KF719143 | NA | NA | NA | 94 | A |
| 144 | KF719144 | *Ascaris* sp. | KF719144 | NA | NA | NA | 18 | C |
| 145 | KF719145 | *Ascaris* sp. | KF719145 | NA | NA | NA | 38 | B |
| 146 | KF719146 | *A. lumbricoides* | KF719146 | NA | NA | NA | 2 | A |
| 147 | KF719147 | *A. lumbricoides* | KF719147 | NA | NA | NA | 2 | A |
| 148 | KF719148 | *A. lumbricoides* | KF719148 | NA | NA | NA | 5 | A |
| 149 | KF719149 | *A. lumbricoides* | KF719149 | NA | NA | NA | 89 | A |
| 150 | KF719150 | *A. lumbricoides* | KF719150 | NA | NA | NA | 89 | A |
| 151 | KM365020 | *A. lumbricoides* | KM365020 | NA | NA | NA | 38 | B |
| 152 | KM365021 | *A. lumbricoides* | KM365021 | NA | NA | NA | 38 | B |
| 153 | KM365023 | *A. lumbricoides* | KM365023 | NA | NA | NA | 38 | B |
| 154 | KX022397 | *A. lumbricoides* | KX022397 | NA | NA | NA | 2 | A |
| 155 | KY045800.1:1-1578 | *A. suum* | KY045800.1:1-1578 | KY045800.1:1-1578 | NA | NA | 5 | A |
| 156 | KY045804.1 | *A. suum* | KY045804.1 | KY045804.1 | NA | NA | 16 | C |
| 157 | KY200852 | *Ascaris* sp. | KY200852 | NA | NA | NA | 5 | A |
| 158 | KY200853 | *Ascaris* sp. | KY200853 | NA | NA | NA | 40 | A |
| 159 | KY200854 | *Ascaris* sp. | KY200854 | NA | NA | NA | 38 | B |
| 160 | KY200855 | *Ascaris* sp. | KY200855 | NA | NA | NA | 2 | A |
| 161 | KY200856 | *Ascaris* sp. | KY200856 | NA | NA | NA | 41 | A |
| 162 | KY200857 | *Ascaris* sp. | KY200857 | NA | NA | NA | 42 | A |
| 163 | KY200858 | *Ascaris* sp. | KY200858 | NA | NA | NA | 43 | A |
| 164 | KY200859 | *Ascaris* sp. | KY200859 | NA | NA | NA | 44 | A |
| 165 | KY200860 | *Ascaris* sp. | KY200860 | NA | NA | NA | 45 | A |
| 166 | KY200861 | *Ascaris* sp. | KY200861 | NA | NA | NA | 46 | A |
| 167 | KY368755.1:43-1578 | *A. lumbricoides* | KY368755.1:43-1578 | KY368755.1:43-1578 | NA | NA | 38 | B |
| 168 | KY368757.1:36-1571 | *A. lumbricoides* | KY368757.1:36-1571 | NA | NA | NA | 38 | B |
| 169 | KY368759.1:43-1578 | *A. lumbricoides* | KY368759.1:43-1578 | KY368759.1:43-1578 | NA | NA | 38 | B |
| 170 | KY368763.1:43-1578 | *A. lumbricoides* | KY368763.1:43-1578 | KY368763.1:43-1578 | NA | NA | 38 | B |
| 171 | KY368764.1:37-1572 | *A. lumbricoides* | KY368764.1:37-1572 | KY368764.1:37-1572 | NA | NA | 38 | B |
| 172 | MF358904 | *A. lumbricoides* | MF358904 | NA | NA | NA | 99 | A |
| 173 | MF358905 | *A. suum* | MF358905 | NA | NA | NA | 5 | A |
| 174 | MF358906 | *A. lumbricoides* | MF358906 | NA | NA | NA | 97 | A |
| 175 | MF358907 | *A. lumbricoides* | MF358907 | NA | NA | NA | 2 | A |
| 176 | MF358908 | *A. lumbricoides* | MF358908 | NA | NA | NA | 100 | B |
| 177 | MF358909 | *A. lumbricoides* | MF358909 | NA | NA | NA | 4 | A |
| 178 | MF358910 | *A. lumbricoides* | MF358910 | NA | NA | NA | 8 | A |
| 179 | MF358911 | *A. suum* | MF358911 | NA | NA | NA | 2 | A |
| 180 | MF358912 | *A. lumbricoides* | MF358912 | NA | NA | NA | 8 | A |
| 181 | MF358913 | *A. lumbricoides* | MF358913 | NA | NA | NA | 98 | A |
| 182 | MF358914 | *A. lumbricoides* | MF358914 | NA | NA | NA | 3 | A |
| 183 | MF358915 | *A. lumbricoides* | MF358915 | NA | NA | NA | 5 | A |
| 184 | MF358916 | *A. lumbricoides* | MF358916 | NA | NA | NA | 8 | A |
| 185 | MF358917 | *A. lumbricoides* | MF358917 | NA | NA | NA | 2 | A |
| 186 | MF358918 | *A. lumbricoides* | MF358918 | NA | NA | NA | 4 | A |
| 187 | MF358919 | *A. lumbricoides* | MF358919 | NA | NA | NA | 5 | A |
| 188 | MF358920 | *A. lumbricoides* | MF358920 | NA | NA | NA | 102 | A |
| 189 | MF358921 | *A. lumbricoides* | MF358921 | NA | NA | NA | 6 | A |
| 190 | MF358922 | *A. suum* | MF358922 | NA | NA | NA | 5 | A |
| 191 | MF358923 | *A. lumbricoides* | MF358923 | NA | NA | NA | 8 | A |
| 192 | MF358924 | *A. lumbricoides* | MF358924 | NA | NA | NA | 3 | A |
| 193 | MF358925 | *A. lumbricoides* | MF358925 | NA | NA | NA | 2 | A |
| 194 | MF358926 | *A. lumbricoides* | MF358926 | NA | NA | NA | 6 | A |
| 195 | MF358927 | *A. lumbricoides* | MF358927 | NA | NA | NA | 101 | A |
| 196 | MF358928 | *A. lumbricoides* | MF358928 | NA | NA | NA | 3 | A |
| 197 | MF358929 | *A. lumbricoides* | MF358929 | NA | NA | NA | 8 | A |
| 198 | MF358930 | *A. lumbricoides* | MF358930 | NA | NA | NA | 2 | A |
| 199 | MF358931 | *A. suum* | MF358931 | NA | NA | NA | 1 | A |
| 200 | MF358932 | *A. lumbricoides* | MF358932 | NA | NA | NA | 3 | A |
| 201 | MF358933 | *A. suum* | MF358933 | NA | NA | NA | 5 | A |
| 202 | MF358934 | *A. lumbricoides* | MF358934 | NA | NA | NA | 12 | A |
| 203 | MF358935 | *A. lumbricoides* | MF358935 | NA | NA | NA | 5 | A |
| 204 | MH795157.1 | *A. suum* | MH795157.1 | NA | NA | NA | 5 | A |
| 205 | MK792795.1 | *A. lumbricoides* | MK792795.1 | NA | NA | NA | 2 | A |
| 206 | MK792798.1 | *A. lumbricoides* | MK792798.1 | NA | NA | NA | 3 | A |
| 207 | MK792800.1 | *A. lumbricoides* | MK792800.1 | NA | NA | NA | 3 | A |
| 208 | MK792806.1 | *A. lumbricoides* | MK792806.1 | NA | NA | NA | 2 | A |
| 209 | MK792809.1 | *A. lumbricoides* | MK792809.1 | NA | NA | NA | 2 | A |
| 210 | MK792811.1 | *A. lumbricoides* | MK792811.1 | NA | NA | NA | 3 | A |
| 211 | MK792812.1 | *A. lumbricoides* | MK792812.1 | NA | NA | NA | 104 | A |
| 212 | MK792813.1 | *A. lumbricoides* | MK792813.1 | NA | NA | NA | 104 | A |
| 213 | MT993838.1 | *A. ovis* isolate Aosc | MT993838.1 | MT993838.1 | NA | NA | 106 | B |
| 214 | MW006083.1 | *A. lumbricoides* | MW006083.1 | NA | NA | NA | 38 | B |
| 215 | MW006084.1 | *A. lumbricoides* | MW006084.1 | NA | NA | NA | 3 | A |
| 216 | MW006085.1 | *A. suum* | MW006085.1 | NA | NA | NA | 5 | A |
| 217 | MW006086.1 | *A. suum* | MW006086.1 | NA | NA | NA | 103 | A |
| 218 | NC_001327.1:8771-10347 | *A. suum* | NC_001327.1:8771-10347 | NC_001327.1:8771-10347 | NA | NA | 5 | A |
| 219 | NC_016198.1:1-1578 | *A. lumbricoides* | NC_016198.1:1-1578 | NC_016198.1:1-1578 | NA | NA | 38 | B |
| 220 | NC_036666.1:1-1577 (KU522453) | *A. ovis* | NC_036666.1:1-1577 (KU522453) | NC_036666.1:1-1577 (KU522453) | NA | NA | 106 | B |
| 221 | X54253.1:8771-10348 | *A. suum* | X54253.1:8771-10348 | X54253.1:8771-10348 | NA | NA | 5 | A |
| 222 | COK_E1 | *A. lumbricoides* | PX991992 | NA | NA | NA | 3 | A |
| 223 | COK_E10 | *A. lumbricoides* | PX991999 | NA | NA | NA | 3 | A |
| 224 | COK_E11 | *A. lumbricoides* | PX992000 | NA | NA | NA | 3 | A |
| 225 | COK_E12 | *A. lumbricoides* | PX992001 | NA | NA | NA | 2 | A |
| 226 | COK_E13 | *A. lumbricoides* | PX992002 | NA | NA | NA | 3 | A |
| 227 | COK_E14 | *A. lumbricoides* | PX992003 | NA | NA | NA | 3 | A |
| 228 | COK_E16 | *A. lumbricoides* | PX992004 | NA | NA | NA | 2 | A |
| 229 | COK_E17 | *A. lumbricoides* | PX992005 | NA | NA | NA | 3 | A |
| 230 | COK_E18 | *A. lumbricoides* | PX992006 | NA | NA | NA | 5 | A |
| 231 | COK_E2 | *A. lumbricoides* | PX991993 | NA | NA | NA | 3 | A |
| 232 | COK_E20 | *A. lumbricoides* | PX992007 | NA | NA | NA | 3 | A |
| 233 | COK_E21 | *A. lumbricoides* | PX992008 | NA | NA | NA | 2 | A |
| 234 | COK_E3 | *A. lumbricoides* | PX991994 | NA | NA | NA | 2 | A |
| 235 | COK_E4 | *A. lumbricoides* | PX991995 | NA | NA | NA | 3 | A |
| 236 | COK_E5 | *A. lumbricoides* | PX991996 | NA | NA | NA | 13 | A |
| 237 | COK_E7 | *A. lumbricoides* | PX991997 | NA | NA | NA | 3 | A |
| 238 | COK_E9 | *A. lumbricoides* | PX991998 | NA | NA | NA | 3 | A |
| 239 | COK_E22 | *A. lumbricoides* | NA | PZ059843 | NA | NA | NA | NA |
| 240 | COK_E23 | *A. lumbricoides* | NA | PZ059844 | NA | NA | NA | NA |
| 241 | COK_E26 | *A. lumbricoides* | NA | PZ059845 | NA | NA | NA | NA |
| 242 | COK_E27 | *A. lumbricoides* | NA | PZ059846 | NA | NA | NA | NA |
| 243 | COK_E28 | *A. lumbricoides* | NA | PZ059847 | NA | NA | NA | NA |
| 244 | COK_E29 | *A. lumbricoides* | NA | PZ059848 | NA | NA | NA | NA |
| 245 | COK_E30 | *A. lumbricoides* | NA | PZ059849 | NA | NA | NA | NA |
| 246 | COK_E31 | *A. lumbricoides* | NA | PZ059850 | NA | NA | NA | NA |
| 247 | COK_E32 | *A. lumbricoides* | NA | PZ059851 | NA | NA | NA | NA |
| 248 | COK_E34 | *A. lumbricoides* | NA | PZ059852 | NA | NA | NA | NA |
| 249 | COK_E35 | *A. lumbricoides* | NA | PZ059853 | NA | NA | NA | NA |
| 250 | COK_E38 | *A. lumbricoides* | NA | PZ059854 | NA | NA | NA | NA |
| 251 | COK_E39 | *A. lumbricoides* | NA | PZ059855 | NA | NA | NA | NA |
| 252 | COK_E41 | *A. lumbricoides* | NA | PZ059856 | NA | NA | NA | NA |
| 253 | COK_E44 | *A. lumbricoides* | NA | NA | PZ208154 | NA | NA | NA |
| 254 | COK_E45 | *A. lumbricoides* | NA | NA | PZ208155 | NA | NA | NA |
| 255 | COK_E46 | *A. lumbricoides* | NA | NA | PZ208156 | NA | NA | NA |
| 256 | COK_E47 | *A. lumbricoides* | NA | NA | PZ208157 | NA | NA | NA |
| 257 | COK_E49 | *A. lumbricoides* | NA | NA | PZ208158 | NA | NA | NA |
| 258 | COK_E50 | *A. lumbricoides* | NA | NA | PZ208159 | NA | NA | NA |
| 259 | COK_E52 | *A. lumbricoides* | NA | NA | PZ208160 | NA | NA | NA |
| 260 | COK_E53 | *A. lumbricoides* | NA | NA | PZ208161 | NA | NA | NA |
| 261 | COK_E54 | *A. lumbricoides* | NA | NA | PZ208162 | NA | NA | NA |
| 262 | COK_E57 | *A. lumbricoides* | NA | NA | PZ208163 | NA | NA | NA |
| 263 | COK_E58 | *A. lumbricoides* | NA | NA | PZ208164 | NA | NA | NA |
| 264 | COK_E60 | *A. lumbricoides* | NA | NA | PZ208165 | NA | NA | NA |
| 265 | COK_E64 | *A. lumbricoides* | NA | NA | NA | PZ137448 | NA | NA |
| 266 | COK_E68 | *A. lumbricoides* | NA | NA | NA | PZ137449 | NA | NA |
| 267 | COK_E69 | *A. lumbricoides* | NA | NA | NA | PZ137450 | NA | NA |
| 268 | COK_E70 | *A. lumbricoides* | NA | NA | NA | PZ137451 | NA | NA |
| 269 | COK_E71 | *A. lumbricoides* | NA | NA | NA | PZ137452 | NA | NA |
| 270 | COK_E74 | *A. lumbricoides* | NA | NA | NA | PZ137453 | NA | NA |
| 271 | COK_E75 | *A. lumbricoides* | NA | NA | NA | PZ137454 | NA | NA |
| 272 | COK_E76 | *A. lumbricoides* | NA | NA | NA | PZ137455 | NA | NA |
| 273 | COK_E80 | *A. lumbricoides* | NA | NA | NA | PZ137456 | NA | NA |
| 274 | COK_E81 | *A. lumbricoides* | NA | NA | NA | PZ137457 | NA | NA |
| 275 | COK1 | *A. lumbricoides* | PX991988 | PX990261 | PZ208150 | PZ137444 | 2 | A |
| 276 | COK2 | *A. lumbricoides* | PX991989 | PX990262 | PZ208151 | PZ137445 | 2 | A |
| 277 | COK3 | *A. lumbricoides* | PX991990 | PX990263 | PZ208152 | PZ137446 | 2 | A |
| 278 | COK4 | *A. lumbricoides* | PX991991 | PX990264 | PZ208153 | PZ137447 | 12 | A |
| 279 | TGK1 | *A. lumbricoides* | OK559746.1 | PX990322 | PZ208135 | PZ137429 | 1 | A |
| 280 | TGK10 | *A. lumbricoides* | OK559755.1 | PX990331 | PZ208144 | PZ137438 | 3 | A |
| 281 | TGK11 | *A. lumbricoides* | OK559756.1 | PX990332 | PZ208145 | PZ137439 | 4 | A |
| 282 | TGK12 | *A. lumbricoides* | OK559757.1 | PX990333 | PZ208146 | PZ137440 | 3 | A |
| 283 | TGK13 | *A. lumbricoides* | OK559758.1 | PX990334 | PZ208147 | PZ137441 | 3 | A |
| 284 | TGK14 | *A. lumbricoides* | OK559759.1 | PX990335 | PZ208148 | PZ137442 | 2 | A |
| 285 | TGK15 | *A. lumbricoides* | OK559760.1 | PX990336 | PZ208149 | PZ137443 | 3 | A |
| 286 | TGK2 | *A. lumbricoides* | OK559747.1 | PX990323 | PZ208136 | PZ137430 | 2 | A |
| 287 | TGK3 | *A. lumbricoides* | OK559748.1 | PX990324 | PZ208137 | PZ137431 | 3 | A |
| 288 | TGK4 | *A. lumbricoides* | OK559749.1 | PX990325 | PZ208138 | PZ137432 | 2 | A |
| 289 | TGK5 | *A. lumbricoides* | OK559750.1 | PX990326 | PZ208139 | PZ137433 | 2 | A |
| 290 | TGK6 | *A. lumbricoides* | OK559751.1 | PX990327 | PZ208140 | PZ137434 | 2 | A |
| 291 | TGK7 | *A. lumbricoides* | OK559752.1 | PX990328 | PZ208141 | PZ137435 | 3 | A |
| 292 | TGK8 | *A. lumbricoides* | OK559753.1 | PX990329 | PZ208142 | PZ137436 | 2 | A |
| 293 | TGK9 | *A. lumbricoides* | OK559754.1 | PX990330 | PZ208143 | PZ137437 | 3 | A |
| 294 | TSL1 | *A. lumbricoides* | OK559761.1 | PX990306 | PZ208092 | PZ137386 | 3 | A |
| 295 | TSL10 | *A. lumbricoides* | OK559769.1 | PX990285 | PZ208101 | PZ137395 | 5 | A |
| 296 | TSL11 | *A. lumbricoides* | OK559770.1 | PX990286 | PZ208102 | PZ137396 | 7 | A |
| 297 | TSL12 | *A. lumbricoides* | OK559771.1 | PX990287 | PZ208103 | PZ137397 | 2 | A |
| 298 | TSL13 | *A. lumbricoides* | OK559772.1 | PX990288 | PZ208104 | PZ137398 | 2 | A |
| 299 | TSL14 | *A. lumbricoides* | OK559773.1 | PX990289 | PZ208105 | PZ137399 | 4 | A |
| 300 | TSL15 | *A. lumbricoides* | OK559774.1 | PX990290 | PZ208106 | PZ137400 | 2 | A |
| 301 | TSL16 | *A. lumbricoides* | OK559775.1 | PX990291 | PZ208107 | PZ137401 | 7 | A |
| 302 | TSL17 | *A. lumbricoides* | OK559776.1 | PX990292 | PZ208108 | PZ137402 | 6 | A |
| 303 | TSL18 | *A. lumbricoides* | OK559777.1 | PX990293 | PZ208109 | PZ137403 | 4 | A |
| 304 | TSL19 | *A. lumbricoides* | OK559778.1 | PX990294 | PZ208110 | PZ137404 | 8 | A |
| 305 | TSL2 | *A. lumbricoides* | OK559762.1 | PX990307 | PZ208093 | PZ137387 | 5 | A |
| 306 | TSL20 | *A. lumbricoides* | OK559779.1 | PX990295 | PZ208111 | PZ137405 | 8 | A |
| 307 | TSL21 | *A. lumbricoides* | OK559780.1 | PX990296 | PZ208112 | PZ137406 | 6 | A |
| 308 | TSL22 | *A. lumbricoides* | OK559781.1 | PX990297 | PZ208113 | PZ137407 | 3 | A |
| 309 | TSL23 | *A. lumbricoides* | OK559782.1 | PX990298 | PZ208114 | PZ137408 | 2 | A |
| 310 | TSL24 | *A. lumbricoides* | OK559783.1 | PX990299 | PZ208115 | PZ137409 | 2 | A |
| 311 | TSL25 | *A. lumbricoides* | OK559784.1 | PX990300 | PZ208116 | PZ137410 | 3 | A |
| 312 | TSL26 | *A. lumbricoides* | OK559785.1 | PX990301 | PZ208117 | PZ137411 | 2 | A |
| 313 | TSL27 | *A. lumbricoides* | OK559786.1 | PX990302 | PZ208118 | PZ137412 | 4 | A |
| 314 | TSL28 | *A. lumbricoides* | OK559787.1 | PX990303 | PZ208119 | PZ137413 | 4 | A |
| 315 | TSL29 | *A. lumbricoides* | OK559788.1 | PX990304 | PZ208120 | PZ137414 | 2 | A |
| 316 | TSL3 | *A. lumbricoides* | OK559763.1 | PX990308 | PZ208094 | PZ137388 | 4 | A |
| 317 | TSL30 | *A. lumbricoides* | OK559789.1 | PX990305 | PZ208121 | PZ137415 | 3 | A |
| 318 | TSL31 | *A. lumbricoides* | OK559790.1 | PX990309 | PZ208122 | PZ137416 | 3 | A |
| 319 | TSL32 | *A. lumbricoides* | OK559791.1 | PX990310 | PZ208123 | PZ137417 | 7 | A |
| 320 | TSL33 | *A. lumbricoides* | OK559792.1 | PX990311 | PZ208124 | PZ137418 | 2 | A |
| 321 | TSL34 | *A. lumbricoides* | OK559793.1 | PX990312 | PZ208125 | PZ137419 | 2 | A |
| 322 | TSL35 | *A. lumbricoides* | OK559794.1 | PX990313 | PZ208126 | PZ137420 | 2 | A |
| 323 | TSL36 | *A. lumbricoides* | PX991943 | PX990314 | PZ208127 | PZ137421 | 2 | A |
| 324 | TSL37 | *A. lumbricoides* | OK559795.1 | PX990315 | PZ208128 | PZ137422 | 2 | A |
| 325 | TSL38 | *A. lumbricoides* | OK559796.1 | PX990316 | PZ208129 | PZ137423 | 8 | A |
| 326 | TSL39 | *A. lumbricoides* | OK559797.1 | PX990317 | PZ208130 | PZ137424 | 3 | A |
| 327 | TSL4 | *A. lumbricoides* | OK559764.1 | PX990279 | PZ208095 | PZ137389 | 3 | A |
| 328 | TSL40 | *A. lumbricoides* | OK559798.1 | PX990318 | PZ208131 | PZ137425 | 4 | A |
| 329 | TSL41 | *A. lumbricoides* | OK559799.1 | PX990319 | PZ208132 | PZ137426 | 4 | A |
| 330 | TSL42 | *A. lumbricoides* | OK559800.1 | PX990320 | PZ208133 | PZ137427 | 4 | A |
| 331 | TSL43 | *A. lumbricoides* | OK559801.1 | PX990321 | PZ208134 | PZ137428 | 5 | A |
| 332 | TSL5 | *A. lumbricoides* | OK559765.1 | PX990280 | PZ208096 | PZ137390 | 3 | A |
| 333 | TSL6 | *A. lumbricoides* | OK559766.1 | PX990281 | PZ208097 | PZ137391 | 4 | A |
| 334 | TSL7 | *A. lumbricoides* | OK559767.1 | PX990282 | PZ208098 | PZ137392 | 6 | A |
| 335 | TSL8 | *A. lumbricoides* | PX991942 | PX990283 | PZ208099 | PZ137393 | 5 | A |
| 336 | TSL9 | *A. lumbricoides* | OK559768.1 | PX990284 | PZ208100 | PZ137394 | 3 | A |
| 337 | TUP1 | *A. lumbricoides* | PX991944 | PX990217 | PZ208048 | PZ137342 | 3 | A |
| 338 | TUP10 | *A. lumbricoides* | PX991953 | PX990226 | PZ208057 | PZ137351 | 5 | A |
| 339 | TUP11 | *A. lumbricoides* | PX991954 | PX990227 | PZ208058 | PZ137352 | 3 | A |
| 340 | TUP12 | *A. lumbricoides* | PX991955 | PX990228 | PZ208059 | PZ137353 | 2 | A |
| 341 | TUP13 | *A. lumbricoides* | PX991956 | PX990229 | PZ208060 | PZ137354 | 3 | A |
| 342 | TUP14 | *A. lumbricoides* | PX991957 | PX990230 | PZ208061 | PZ137355 | 3 | A |
| 343 | TUP15 | *A. lumbricoides* | PX991958 | PX990231 | PZ208062 | PZ137356 | 9 | A |
| 344 | TUP16 | *A. lumbricoides* | PX991959 | PX990232 | PZ208063 | PZ137357 | 2 | A |
| 345 | TUP17 | *A. lumbricoides* | PX991960 | PX990233 | PZ208064 | PZ137358 | 2 | A |
| 346 | TUP18 | *A. lumbricoides* | PX991961 | PX990234 | PZ208065 | PZ137359 | 2 | A |
| 347 | TUP19 | *A. lumbricoides* | PX991962 | PX990235 | PZ208066 | PZ137360 | 2 | A |
| 348 | TUP2 | *A. lumbricoides* | PX991945 | PX990218 | PZ208049 | PZ137343 | 2 | A |
| 349 | TUP20 | *A. lumbricoides* | PX991963 | PX990236 | PZ208067 | PZ137361 | 10 | A |
| 350 | TUP21 | *A. lumbricoides* | PX991964 | PX990237 | PZ208068 | PZ137362 | 9 | A |
| 351 | TUP22 | *A. lumbricoides* | PX991965 | PX990238 | PZ208069 | PZ137363 | 2 | A |
| 352 | TUP23 | *A. lumbricoides* | PX991966 | PX990239 | PZ208070 | PZ137364 | 9 | A |
| 353 | TUP24 | *A. lumbricoides* | PX991967 | PX990240 | PZ208071 | PZ137365 | 9 | A |
| 354 | TUP25 | *A. lumbricoides* | PX991968 | PX990241 | PZ208072 | PZ137366 | 5 | A |
| 355 | TUP26 | *A. lumbricoides* | PX991969 | PX990242 | PZ208073 | PZ137367 | 11 | A |
| 356 | TUP27 | *A. lumbricoides* | PX991970 | PX990243 | PZ208074 | PZ137368 | 3 | A |
| 357 | TUP28 | *A. lumbricoides* | PX991971 | PX990244 | PZ208075 | PZ137369 | 3 | A |
| 358 | TUP29 | *A. lumbricoides* | PX991972 | PX990245 | PZ208076 | PZ137370 | 3 | A |
| 359 | TUP3 | *A. lumbricoides* | PX991946 | PX990219 | PZ208050 | PZ137344 | 2 | A |
| 360 | TUP30 | *A. lumbricoides* | PX991973 | PX990246 | PZ208077 | PZ137371 | 9 | A |
| 361 | TUP31 | *A. lumbricoides* | PX991974 | PX990247 | PZ208078 | PZ137372 | 3 | A |
| 362 | TUP32 | *A. lumbricoides* | PX991975 | PX990248 | PZ208079 | PZ137373 | 3 | A |
| 363 | TUP33 | *A. lumbricoides* | PX991976 | PX990249 | PZ208080 | PZ137374 | 9 | A |
| 364 | TUP34 | *A. lumbricoides* | PX991977 | PX990250 | PZ208081 | PZ137375 | 3 | A |
| 365 | TUP35 | *A. lumbricoides* | PX991978 | PX990251 | PZ208082 | PZ137376 | 3 | A |
| 366 | TUP36 | *A. lumbricoides* | PX991979 | PX990252 | PZ208083 | PZ137377 | 2 | A |
| 367 | TUP37 | *A. lumbricoides* | PX991980 | PX990253 | PZ208084 | PZ137378 | 2 | A |
| 368 | TUP38 | *A. lumbricoides* | PX991981 | PX990254 | PZ208085 | PZ137379 | 9 | A |
| 369 | TUP39 | *A. lumbricoides* | PX991982 | PX990255 | PZ208086 | PZ137380 | 9 | A |
| 370 | TUP4 | *A. lumbricoides* | PX991947 | PX990220 | PZ208051 | PZ137345 | 2 | A |
| 371 | TUP40 | *A. lumbricoides* | PX991983 | PX990256 | PZ208087 | PZ137381 | 9 | A |
| 372 | TUP41 | *A. lumbricoides* | PX991984 | PX990257 | PZ208088 | PZ137382 | 11 | A |
| 373 | TUP42 | *A. lumbricoides* | PX991985 | PX990258 | PZ208089 | PZ137383 | 3 | A |
| 374 | TUP43 | *A. lumbricoides* | PX991986 | PX990259 | PZ208090 | PZ137384 | 5 | A |
| 375 | TUP44 | *A. lumbricoides* | PX991987 | PX990260 | PZ208091 | PZ137385 | 3 | A |
| 376 | TUP5 | *A. lumbricoides* | PX991948 | PX990221 | PZ208052 | PZ137346 | 3 | A |
| 377 | TUP6 | *A. lumbricoides* | PX991949 | PX990222 | PZ208053 | PZ137347 | 9 | A |
| 378 | TUP7 | *A. lumbricoides* | PX991950 | PX990223 | PZ208054 | PZ137348 | 10 | A |
| 379 | TUP8 | *A. lumbricoides* | PX991951 | PX990224 | PZ208055 | PZ137349 | 9 | A |
| 380 | TUP9 | *A. lumbricoides* | PX991952 | PX990225 | PZ208056 | PZ137350 | 3 | A |

NA: not available; TGK: Tha Song Yang District, Tak Province (Greh-kee locality); TSL: Tha Song Yang District, Tak Province (Salid Luang locality); TUP: Umphang District, Tak Province; COK: Omkoi District, Chiang Mai Province; COK_E: Omkoi District, Chiang Mai Province (genomic DNA was extracted from a single egg).
